# Pre-Deployment Audit of Actionability and Equity in Severe Tooth Loss Prediction Using Constrained Algorithmic Recourse and Temporal Validation

**DOI:** 10.3390/bioengineering13091020

**Published:** 2026-09-01

**Authors:** Quang Tuan Lam, Fang-Yu Fan, Tzu-Yu Peng, Sheng-Wei Feng, Cathy Chia-Yu Huang, Tong-Hsien Chow, Minh Huu Nhat Le, Giang Vu, Yung-Li Wang, Nguyen Quoc Khanh Le, I-Ta Lee

**Affiliations:** 1 School of Dentistry, College of Oral Medicine, Taipei Medical University, Taipei 11031, Taiwan; d204111005@tmu.edu.tw (Q.T.L.); typeng@tmu.edu.tw (T.-Y.P.); shengwei@tmu.edu.tw (S.-W.F.); 2 AIBioMed Research Group, Taipei Medical University, Taipei 11031, Taiwan; 3 Faculty of Odonto-Stomatology, Da Nang University of Medical Technology and Pharmacy, Da Nang 550000, Vietnam; 4 School of Dental Technology, College of Oral Medicine, Taipei Medical University, Taipei 11031, Taiwan; fish884027@tmu.edu.tw; 5 Department of Life Sciences, National Central University, Taoyuan City 32001, Taiwan; chuang@ncu.edu.tw; 6 Department of Sports Science, R.O.C. Military Academy, Kaohsiung 83059, Taiwan; thchowma@gmail.com; 7 Section of Cardiovascular Medicine, Department of Internal Medicine, Yale School of Medicine, Yale University, New Haven, CT 06520, USA; d142111009@tmu.edu.tw; 8 School of Global Health Management and Informatics, University of Central Florida, Orlando, FL 32801, USA; giang.vu@ucf.edu; 9 Artificial Intelligence in Medicine, College of Medicine, Taipei Medical University, Taipei 11031, Taiwan; 10 Translational Imaging Research Center, Taipei Medical University Hospital, Taipei 11031, Taiwan

**Keywords:** severe tooth loss, algorithmic recourse, artificial intelligence, health equity, temporal validation

## Abstract

Background: Predictive performance does not establish whether model-identified risks correspond to feasible, equitable pathways. We assessed the actionability and equity of a severe tooth loss prediction model using constrained algorithmic recourse and same-source temporal validation. Methods: An Explainable Boosting Machine was trained using 2022 Behavioral Risk Factor Surveillance System data from 433,772 adults. High-risk adults underwent recourse auditing incorporating immutability locks, behavioral directionality, physiological safety floors, and at most three feature changes. Recourse represented hypothetical movement within model space, not treatment advice, causal risk reduction, or reversal of tooth loss. Primary reachability was the unweighted analytic-cohort proportion for which the frozen engine identified a feasible pathway; a BRFSS survey-weighted domain sensitivity analysis used final weights, strata, and primary sampling units. The audit separated reachability from conditional burden among reachable adults. Equity was evaluated across Social Indicators of Disparity Index (SIDI) and income strata using Oaxaca–Blinder decomposition. The frozen specification was evaluated in a 2022 holdout and the 2024 BRFSS cohort (*N* = 448,213) without retraining. Results: Survey-weighted areas under the receiver operating characteristic curve were 0.858, 0.855, and 0.858 in the 2022 full, 2022 holdout, and 2024 cohorts. Primary unweighted reachability was 10.61%, 10.54%, and 9.82%; corresponding survey-weighted estimates were 12.69% (95% design-aware CI, 12.33–13.07%), 12.31% (11.54–13.12%), and 11.30% (10.95–11.65%), respectively. Thus, reachability remained limited under both estimands. The high-SIDI group showed poorer calibration. Under the prespecified primary SIDI-neutral additive-cost specification, residual conditional cost differences across SIDI strata were small; alternative burden metrics were direction-dependent. The income residual attenuated from the 2022 holdout to 2024, although its confidence intervals were fixed-pipeline row-bootstrap intervals rather than fully design-based intervals. Conclusions: Stable predictive performance masked limited model-space actionability. Integrating explicitly labeled reachability estimands, conditional burden, equity, and temporal transport can strengthen pre-deployment evaluation. Because severe tooth loss is irreversible, recourse pathways should be interpreted as a stress test of model-implied modifiable factors rather than evidence of reversibility.

## 1. Introduction

Health systems are increasingly adopting artificial intelligence (AI) and machine learning (ML) risk models to support screening, triage, prevention, and population health management [1,2]. Although discrimination, calibration, and temporal validation are essential for evaluating predictive performance, these metrics do not determine whether a high-risk classification can be translated into feasible, equitable, and operationally meaningful action before deployment [3,4]. A model may remain statistically stable across time yet still fail to provide plausible pathways that patients, clinicians, or health systems can act upon. This deployment-readiness gap motivates pre-deployment recourse auditing as a descriptive governance procedure for quantifying model actionability, conditional burden, and pathway composition before any patient-facing workflow is implemented [5,6].

Algorithmic recourse provides a framework for identifying feature changes that would move an individual below an adverse model threshold [7,8,9]. In health applications, however, unconstrained counterfactual explanations may be clinically inappropriate or misleading [8]. A dependable clinical recourse audit should therefore encode domain guardrails, including immutability locks for demographic and socioeconomic characteristics, behavioral directionality to preserve plausible change trajectories, physiological safety floors to prevent unsafe candidate actions, and sparsity limits to avoid unrealistic action bundles [7,8,9]. Under these constraints, recourse should be interpreted as hypothetical model-state movement rather than treatment recommendation, causal risk reduction, or clinical reversal of an existing disease state.

In this study, we propose a locked two-stage pre-deployment audit framework for health risk models. First-stage reachability identifies whether any feasible model-state pathway exists for a high-risk adult under a prespecified action ontology, whereas second-stage conditional burden estimates the minimum model-movement cost only among reachable adults. To summarize equity-axis differences in this second stage, Oaxaca–Blinder decomposition is used as a descriptive accounting tool that partitions conditional cost gaps into a component associated with included baseline covariates and a residual conditional cost difference not accounted for by those covariates [10,11].

We empirically evaluated this framework using severe tooth loss in the Behavioral Risk Factor Surveillance System (BRFSS) [12,13]. Severe tooth loss is an informative oral health outcome for evaluating model actionability because it is socially patterned, behaviorally mediated, measurable in large surveillance cohorts, and irreversible [14,15]. This irreversibility makes the setting particularly appropriate for clarifying the boundary between model-state actionability and clinical intervention: the audit asks whether a locked risk model can surface feasible pathways below an operating threshold, not whether tooth loss can be reversed or causal risk can be reduced. By applying the same locked audit specification across BRFSS cycles, this study evaluates whether predictive stability, reachability, conditional burden, and equity signals transport over time before recourse-enabled decision support is considered.

**Figure 1:**
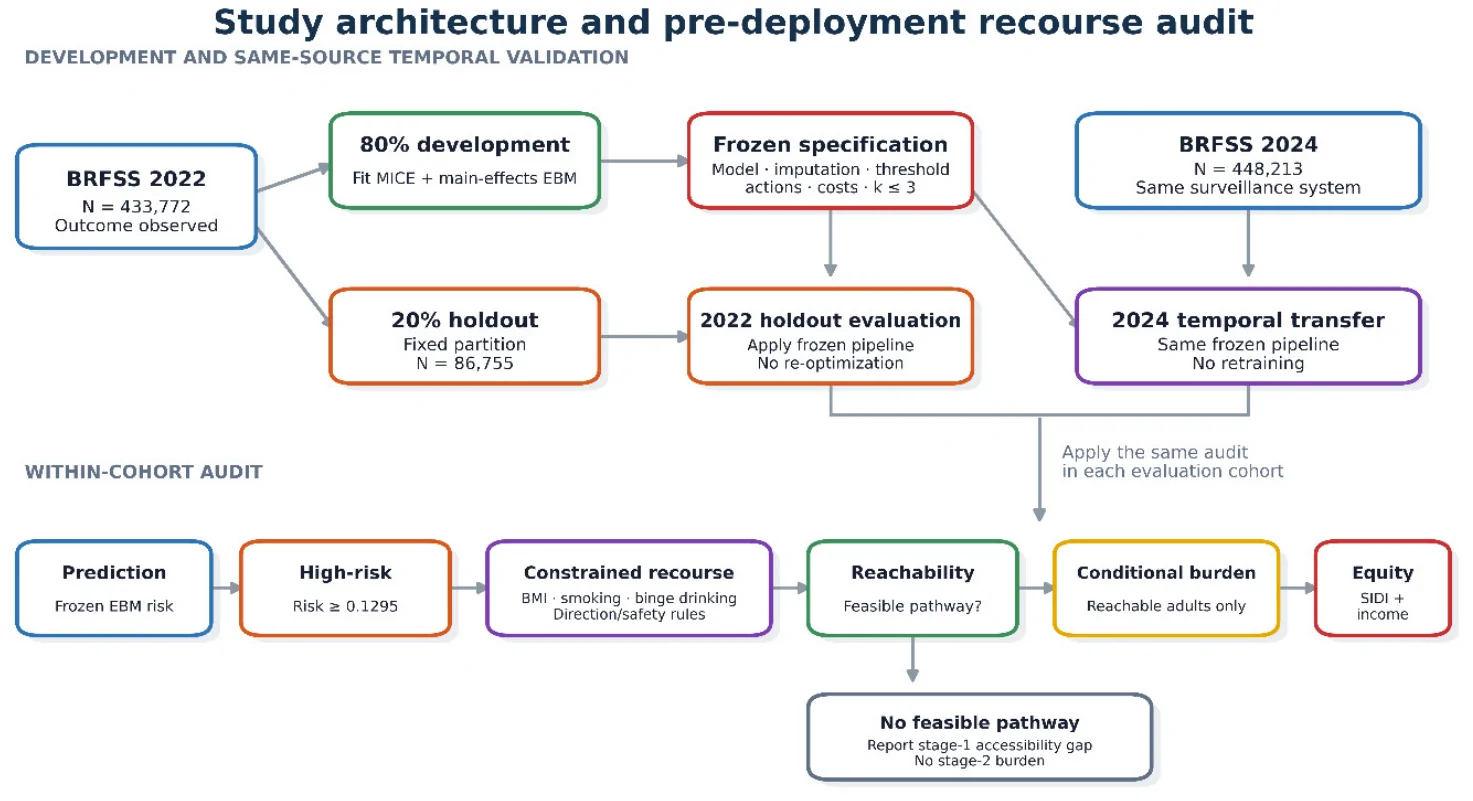
Study architecture and within-cohort recourse-audit flow. The upper section shows the 2022 development/holdout split, frozen imputation/model/threshold/action/cost specifications, 2022 holdout evaluation, and same-source 2024 transfer without retraining. The lower section shows prediction, high-risk classification, constrained recourse, reachability, conditional burden among reachable adults, and equity assessment; the unreachable branch reports the stage-1 accessibility gap. Primary reachability is unweighted; population sensitivity uses survey-weighted domain estimates with design-aware confidence intervals; conditional burden is survey-weighted among reachable adults. BRFSS indicates Behavioral Risk Factor Surveillance System; EBM, Explainable Boosting Machine; SIDI, Social Indicators of Disparity Index. Arrows indicate the workflow and progression between analysis stages; colors are used only for visual distinction and do not encode additional information.

Accordingly, this study aimed to assess the actionability and equity of a severe tooth loss prediction model using a locked two-stage algorithmic recourse framework and temporal validation in BRFSS data. The framework integrates clinically constrained counterfactual search, survey-weighted model evaluation, multi-axis equity stratification, Oaxaca–Blinder decomposition, frozen cross-cycle transport testing, and implementation-facing governance summaries. Specifically, we evaluated whether the framework could: (1) separate first-stage reachability, defined as whether any feasible model-state pathway exists, from second-stage conditional burden, defined as the minimum model-movement cost among reachable adults; (2) transport a locked recourse-audit specification from BRFSS 2022 to BRFSS 2024 without retraining; and (3) determine whether recourse-related equity findings are axis-specific and metric-dependent. These outputs were designed to support pre-deployment review of oral health risk prediction models before any recourse-enabled decision-support workflow is considered for local clinical validation. The locked derivation, holdout evaluation, and frozen temporal-transfer design are summarized in Figure 1.

## 2. Methods

### 2.1. Study Design and Data Sources

This retrospective pre-deployment audit used Centers for Disease Control and Prevention Behavioral Risk Factor Surveillance System (BRFSS) data from 2022 and 2024 [12,13]. The target outcome was severe tooth loss, defined as having ≥6 missing natural teeth; records with missing outcome data were excluded. The 2022 baseline cohort (*N* = 433,772) was stratified by outcome into training (80%) and holdout (20%) partitions. Missing predictors were imputed using a chained-equations framework, with imputation parameters learned strictly from the training set and applied forward to prevent data leakage [16]. To support reproducibility and temporal transport evaluation, the complete audit pipeline was locked before validation. The locked specification included the adverse-risk threshold, recourse cost weights, feasible-action rules, and sparsity constraints. This design prevented post hoc re-optimization of the audit specification based on validation or equity results. For same-source temporal validation, the frozen 2022 specification was applied directly to the 2024 BRFSS cohort (*N* = 448,213) without model retraining. Predictive-performance point estimates incorporated final BRFSS survey weights. Primary reachability was defined as the unweighted analytic-cohort proportion of high-risk records for which the frozen recourse engine found a feasible threshold-crossing pathway. This estimand quantifies computational audit coverage in the analyzed cohort, gives each processed record one audit opportunity, and reconciles directly with the saved high-risk and recourse counts; it is not a design-based population prevalence. As a population-oriented sensitivity analysis, reachability was also estimated as a survey-weighted domain proportion from the full analytic cohort using WEIGHT, STRATA, and PSU, with high-risk status defining the survey domain, Taylor-linearized logit confidence intervals, and centered singleton-PSU adjustment. The 2022 holdout design-aware interval is conditional on the fixed holdout partition and frozen upstream pipeline. BRFSS data are de-identified and publicly available; therefore, this study was determined to be exempt from Institutional Review Board oversight under 45 CFR §46.104(d)(4). Source data are publicly available from the CDC BRFSS. Study-specific derived analytic files, trained models, recourse outputs, and analysis code are archived on Zenodo at https://doi.org/10.5281/zenodo.20783950 (accessed on 27 August 2026).

### 2.2. Model Development and Evaluation

We trained a separate main-effects Explainable Boosting Machine (EBM), a transparent machine learning architecture [17], using the same 19 core predictors examined in our related severe tooth-loss risk stratification study [18]. That prior study developed and externally evaluated a survey-weighted MICE-EBM prediction framework using BRFSS 2022 derivation, BRFSS 2024 same-source temporal validation, and NHANES 2015–2018 cross-survey transportability. Its prediction-model artifacts used 38 inputs, including missingness indicators, and allowed pairwise interaction terms. In contrast, the present study re-specified a distinct 19-feature, main-effects-only 2022 EBM audit artifact to support additive feature-level recourse accounting. Thus, the overlap is limited to the disease endpoint, BRFSS source cohorts, and core predictor domain, whereas the analytic estimand, model constraint, and contribution are different.

The EBM computes risk on the logit scale as an additive sum of feature functions; locked hyperparameters and the prespecified operating threshold are detailed in Supplementary Table S1:

$$g\left(E\left[Y|x\right]\right)=\beta_{0}+\sum_{j}f_{j}\left(x_{j}\right)$$

where g is the logit link, $\beta_{0}$ is the intercept, and $f_{j}$ is the learned shape function for predictor j. This formulation allows each hypothetical feature movement to be mapped to a specific learned risk contribution, preserving feature-level interpretability for the recourse audit.

An adverse-risk threshold (*τ* = 0.1295; reported as 0.130 when rounded to three decimal places) was prespecified on the 2022 training data by maximizing the survey-weighted Youden index [19]. Adults with predicted risk at or above τ were classified as high-risk candidates for recourse-governance analysis:

$$\tau =\arg\underset{t}{\max}\left[\mathrm{Sensitivity}\left(t\right)+\mathrm{Specificity}\left(t\right)-1\right]$$

Predictive performance point estimates were quantified using the final BRFSS survey weight for the holdout area under the receiver operating characteristic curve (AUC) and Brier score [20]. These are survey-weighted descriptive prediction metrics; the reported fixed-model row-bootstrap intervals retained the final weights but did not encode the BRFSS stratification or clustering structure. We distinguished the overall holdout Brier score from the high-risk subset Brier score to assess probability reliability above the operating threshold.

### 2.3. Equity Stratification

Because public BRFSS files lack granular geographic identifiers for direct linkage to area-level deprivation measures such as the CDC Social Vulnerability Index [21], we constructed the Social Indicators of Disparity Index (SIDI) as a study-defined, individual-level audit stratifier. SIDI used four BRFSS-available indicators: long-term unemployment or inability to work, renting rather than owning housing, delayed routine checkups, and high household competing demands. Conceptually, SIDI follows the formative logic of the Townsend Deprivation Index [22] and is aligned with the Social Determinants of Health framework [23]. SIDI was used as a prespecified audit axis rather than as a newly validated deprivation index.

Income and SIDI were treated as distinct audit axes because they capture different dimensions of socioeconomic disadvantage. Component definitions are provided in Supplementary Table S2, and cohort score distributions are summarized in Supplementary Table S3.

$$SIDI_{i}=\sum_{j=1}^{4}I\left(v_{ij}=1\right)$$

where $v_{ij}$ denotes the presence of the deprivation indicator j for individual i. The primary SIDI contrast compared adults with SIDI ≥ 2 against those scoring 0–1.

SIDI was prespecified as a formative, study-constructed individual-level deprivation proxy for audit stratification rather than as a substitute for geocoded area-deprivation indices. Because its components represent distinct deprivation domains rather than interchangeable items on a reflective psychometric scale, construct checks focused on known-groups gradients, component and cutpoint robustness, and temporal missing-component bounds.

To reduce overlap between the predictive and auditing layers, socioeconomic covariates already designated as baseline model features, specifically educational attainment and direct cost barriers to care, were excluded from the SIDI composite. This separation reduces analyst-induced overlap between the audit stratification axis and the downstream cost specification.

### 2.4. Constrained Recourse and Cost Specification

To operationalize algorithmic recourse as a pre-deployment audit, the recourse engine identified sparse hypothetical model-state transitions for each high-risk adult that would move the predicted risk below the adverse threshold under prespecified constraints [9]. Predictor roles, actionability classes, expected time horizon, directionality, reversibility, actor locus, and causal-assumption status are detailed in Supplementary Table S4. Because severe tooth loss is an irreversible prevalent outcome in BRFSS, crossing the model threshold should not be interpreted as reversal of tooth loss or as evidence that actual clinical risk would decrease by the same amount.

Formally, the recourse optimization problem was modeled as:

$$x^{*}=\arg\underset{x^{\prime }}{\min}C\left(x,x^{\prime }\right)\;\mathrm{subject}\;\mathrm{to}\;x^{\prime }\in F\left(x\right),\;f\left(x^{\prime }\right)<\tau ,\;\mathrm{and}\;|x^{\prime }-x{|}_{0}\leq k$$

where $F\left(x\right)$ represents the clinically feasible action space governed by three domain-specific constraint classes:

(1) C1 Immutability Locks: Fixed EBM predictors, including age, sex, income, education, and marital status, were locked against modification.

(2) C2 Behavioral Directionality: Lifestyle variables were restricted to plausible trajectories, such as allowing a current smoker to move to a former-smoker state while preventing retroactive movement to a lifetime never-smoker state.

(3) C3 Physiological Safety Floors: Biological variables were bounded by safety constraints, including capping BMI reduction at the WHO underweight threshold of 18.5 kg/m^2^ [24].

The sparsity parameter k limited the number of feature changes permitted in a candidate pathway. Adults failing to cross τ within this restricted search space were classified as unreachable. We used k ≤ 3 to allow moderate multi-target pathways while avoiding extreme or unrealistic action bundles, consistent with the actionability logic of recourse and evidence that coordinated multiple-behavior change can support feasible intervention design [25,26].

The primary burden outcome was quantified using an additive, SIDI-neutral normalized cost function [9]:

$$C\left(x,x^{\prime }\right)=\sum_{j}w_{j}d\left(x_{j},x_{j}^{\prime }\right)$$

where $w_{j}$ is the feature difficulty weight and d represents the normalized movement for feature j. To ground the audit in epidemiological feasibility, empirical difficulty weights for binary behavioral features were defined as:

$$w_{j}=1-p_{j}$$

where $p_{j}$ denotes the survey-weighted prevalence of the successful target state. Costs were defined inversely to prevalence to operationalize density-aware algorithmic actionability, following the FACE principle that feasible recourse should respect empirical population density [27]. BMI movement was scaled by EBM bin transitions, yielding a neutral engineering estimand of base feature movement under physical constraints [28].

### 2.5. Decomposition and Validation Analyses

Among reachable adults, mean minimum recourse cost disparities were partitioned using a two-fold Oaxaca–Blinder decomposition [10,11].

$$\Delta \bar{Y}=\left(\bar{X_{B}}-\bar{X_{A}}\right)\beta_{A}+\bar{X_{B}}\left(\beta_{B}-\beta_{A}\right)$$

Primary decomposition covariates included age, sex, BMI, smoking status, and race/ethnicity. Race/ethnicity was used only as an equity/decomposition covariate and was not one of the 19 EBM predictors. Decomposition analyses were evaluated across SIDI (≥2 vs. 0–1) and income (categories 1–2 vs. 5–6) axes. The first term represents the endowment component associated with included baseline clinical profiles, whereas the second term estimates the residual conditional cost difference not accounted for by the included audit covariates. Because this analysis was conditioned strictly on reachability, the residual may reflect omitted variables, model specification, reference-group selection, measurement error, cost definition, and selection into the reachable subgroup. It should not be interpreted as a causal estimate of discrimination, intentional bias, or intrinsic unfairness. In this governance application, “unexplained” refers only to statistical disparities not accounted for by the empirical covariates included in the descriptive audit.

Validation analyses used the locked 2022 full-sample artifact, a 2022 holdout rerun, and frozen 2024 lower- and upper-bound SIDI transfer reruns. Inverse-probability-weighted (IPW) analyses [29] and alternative threshold scans were treated as supplemental diagnostics (Supplementary Table S5). Confidence intervals for survey-weighted AUC and Brier scores used 1000 fixed-model individual-row bootstrap replicates with final survey weights. Oaxaca–Blinder point estimates used survey-weighted least squares among reachable adults, and their confidence intervals used 1000 individual-row case-resampling replicates conditional on the fixed upstream model, threshold, and recourse pipeline. These row-bootstrap procedures did not preserve STRATA or PSU structure and therefore are not fully design-based BRFSS confidence intervals; they also omit imputation, model-training, threshold-selection, and recourse-generation uncertainty. Separately, Supplementary Table S6 reports design-aware confidence intervals for survey-weighted reachability using the full survey design and high-risk domain estimation. The Oaxaca–Blinder and bootstrap specifications are detailed in Supplementary Table S7.

We additionally ran positive-control simulations that deliberately inflated conditional cost or restricted an action for monitored groups to verify that the audit responded to injected prescriptive inequity. Finally, audit metrics were packaged into a Stratum-Specific Recourse Cost Profile (SSRCP) for governance reporting.

SIDI-specific robustness diagnostics included lower- and upper-bound 2024 missing-component coding (Supplementary Table S8), alternative binary cutpoints (Supplementary Table S9), and component-level decomposition (Supplementary Table S10). These diagnostics were interpreted as construct and robustness evidence for an audit stratifier, not as external validation of SIDI.

This study was reported with attention to the Transparent Reporting of a multivariable prediction model for Individual Prognosis Or Diagnosis + Artificial Intelligence (TRIPOD + AI) statement [30] and the Strengthening the Reporting of Observational Studies in Epidemiology (STROBE) guidelines [31].

## 3. Results

### 3.1. Cohort Profile and Subgroup Calibration

After outcome exclusions, the 2022 baseline cohort included 433,772 adults, of whom 167,499 had predicted risk ≥0.1295 and entered the recourse-audit cohort. Baseline characteristics of the high-risk cohort supported the known-groups interpretation of the study-constructed SIDI audit stratifier. Compared with low-SIDI adults, high-SIDI adults had lower income, lower educational attainment, lower insurance coverage, more frequent cost-related care barriers, and worse self-rated general health. They also had distinct clinical and behavioral profiles, including younger age, higher BMI, higher current smoking prevalence, and greater severe tooth-loss prevalence (Table 1).

Low-SIDI adults had a Brier score of 0.196, expected calibration error (ECE) of 0.014, and calibration slope of 0.913, whereas high-SIDI adults had a Brier score of 0.208, ECE of 0.035, and calibration slope of 0.798. Thus, subgroup calibration was reported separately from recourse reachability and conditional burden and was incorporated into the governance profile below.

### 3.2. Model Performance and Reachability

The 2022 holdout evaluation showed stable predictive performance, with weighted AUC of 0.855 (95% CI 0.847 to 0.863), weighted Brier score of 0.088 (95% CI 0.085 to 0.091), high-risk subset Brier score of 0.197, calibration slope of 0.999, and ECE of 0.006. However, only 3536 of 33,537 high-risk adults were reachable under the constrained audit specification (10.54%). Temporal transfer to the 2024 cohort (*N* = 448,213) showed minimal same-source performance decay, with a weighted AUC of 0.858 (95% CI 0.854 to 0.861), a weighted Brier score of 0.086 (95% CI 0.084 to 0.087), a calibration slope of 1.014, and ECE of 0.006.

**Figure 2:**
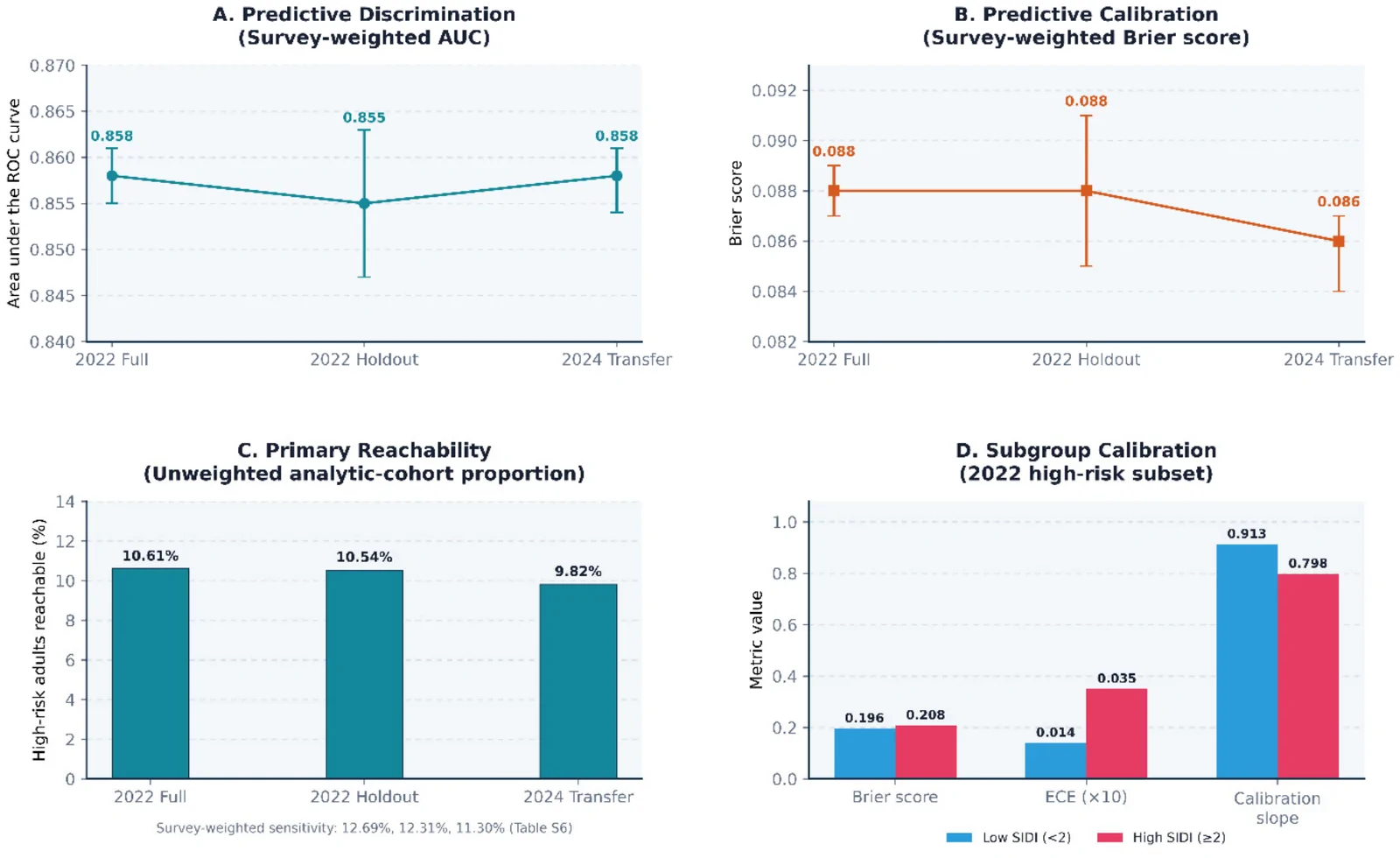
Predictive performance, constrained reachability, and subgroup calibration across validation stages. Panels (**A**,**B**) show final-survey-weighted AUC and Brier point estimates; error bars are 95% fixed-model weighted individual-row bootstrap intervals. Because resampling did not preserve BRFSS STRATA or PSU structure, these intervals are not fully design-based. Panel (**C**) reports primary unweighted analytic-cohort reachability of 10.61%, 10.54%, and 9.82% in the 2022 full artifact, 2022 holdout, and 2024 frozen transfer cohorts; corresponding survey-weighted domain sensitivities were 12.69%, 12.31%, and 11.30% (Supplementary Table S6). Panel (**D**) shows subgroup calibration among the 2022 high-risk subset, with higher Brier score, higher expected calibration error, and lower calibration slope among high-SIDI adults than low-SIDI adults. ECE bars are displayed at 10× scale for visibility; numeric labels show unscaled ECE values. AUC indicates area under the receiver operating characteristic curve; ECE, expected calibration error; PSU, primary sampling unit; SIDI, Social Indicators of Disparity Index. In Panel (**D**), blue indicates the low-SIDI group (<2) and red indicates the high-SIDI group (≥2); colors in Panels (**A**–**C**) are used only for visual distinction and do not encode additional categories.

Constrained reachability remained low and similar across validation stages. The primary unweighted analytic-cohort estimates were 17,766 of 167,499 high-risk adults in the 2022 full artifact (10.61%), 3536 of 33,537 in the 2022 holdout (10.54%), and 17,152 of 174,664 in the frozen 2024 transfer cohort (9.82%) (Table 2, Panel A). Corresponding BRFSS survey-weighted domain estimates were 12.69% (95% design-aware CI, 12.33–13.07%), 12.31% (11.54–13.12%), and 11.30% (10.95–11.65%), respectively (Supplementary Table S6). The survey-weighted estimates were 2.09, 1.76, and 1.48 percentage points higher than the corresponding unweighted analytic-cohort estimates, but both estimands supported the conclusion of limited reachability. Thus, stable predictive performance under same-source temporal transport did not translate into broad model-state actionability, and subgroup calibration differences were also evident among high-risk adults (Figure 2).

**Figure 3:**
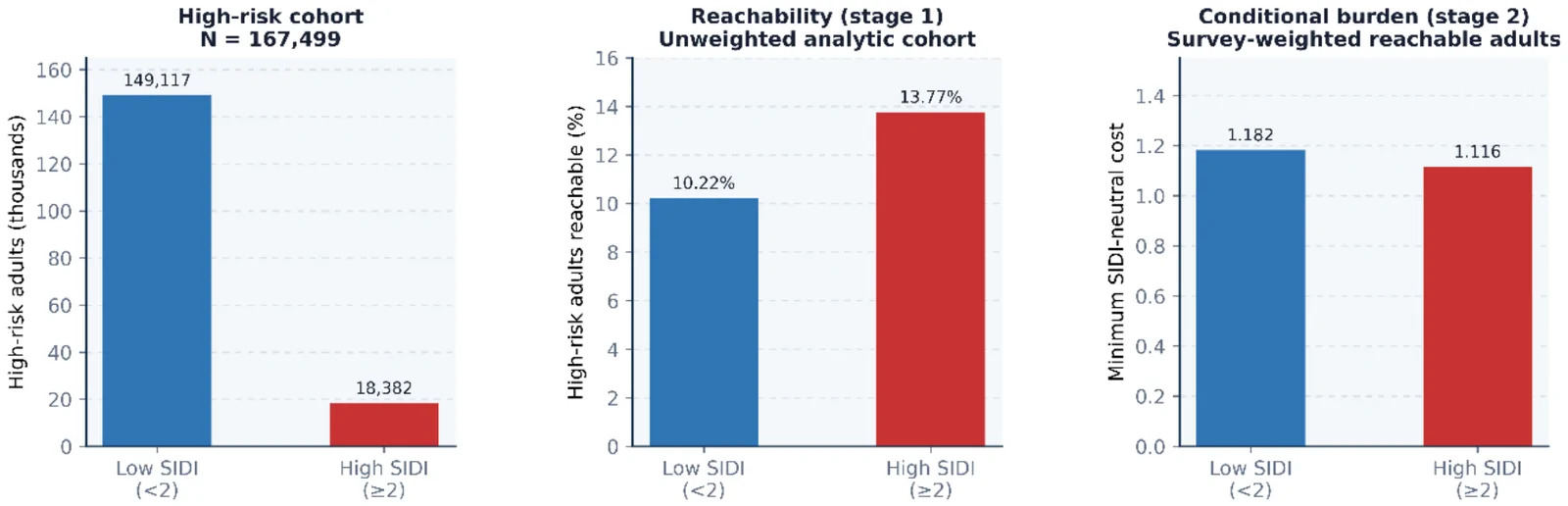
Stratum-specific high-risk cohort size, first-stage reachability, and second-stage conditional burden by SIDI stratum in the 2022 full artifact. The low-SIDI group accounted for 149,117 of 167,499 high-risk adults, whereas the high-SIDI group included 18,382. Primary unweighted analytic-cohort reachability was higher among high-SIDI adults than low-SIDI adults (13.77% vs. 10.22%). Among reachable adults, the survey-weighted mean minimum SIDI-neutral normalized recourse cost was lower in the high-SIDI group than the low-SIDI group (1.116 vs. 1.182). These panels illustrate that cohort size, primary reachability, and conditional burden are distinct audit quantities. SIDI indicates Social Indicators of Disparity Index. Blue indicates the low-SIDI group (<2), and red indicates the high-SIDI group (≥2).

Reachable and unreachable high-risk adults differed in their clinical and behavioral characteristics. Compared with unreachable adults, reachable adults were younger, had higher BMI, were more frequently current smokers, and had lower predicted risk and lower baseline severe tooth-loss prevalence (Table 2, Panel B). Stratum-specific high-risk counts, reachability, and SIDI-neutral mean model-movement costs are summarized in Figure 3.

### 3.3. Primary Neutral-Cost Decomposition

Among reachable adults, the prespecified primary SIDI-neutral additive-cost decomposition showed small residual conditional cost differences across validation stages. The SIDI residual was 0.013 in the 2022 full artifact, 0.025 in the 2022 holdout cohort (95% fixed-pipeline row-bootstrap interval, −0.035 to 0.086), and 0.007 and 0.005 in the 2024 lower- and upper-bound SIDI transfer analyses, respectively (Table 3, Panel A). These results apply to the primary additive-cost metric; alternative burden metrics differed in direction (Supplementary Table S11).

Income-axis residual point estimates were larger: 0.080 in the 2022 full artifact, 0.108 in the 2022 holdout cohort (95% fixed-pipeline weighted row-bootstrap interval, 0.013 to 0.202), and 0.045 in the 2024 transfer cohort (95% fixed-pipeline weighted row-bootstrap interval, −0.001 to 0.095). The 2022 holdout interval excluded zero, whereas the 2024 transfer interval crossed zero marginally. Because the row bootstrap retained weights but did not preserve BRFSS strata or PSU structure, these intervals are descriptive fixed-pipeline uncertainty summaries rather than fully design-based tests. The income-axis conditional-cost signal is therefore described as directionally positive but attenuated in 2024 (Table 3, Panel A).

The small SIDI residual was consistent with supplemental robustness checks. Lower- versus upper-bound missing-component coding in 2024 produced nearly identical estimates (Supplementary Table S8), and alternative SIDI cutpoints preserved the qualitative interpretation of limited SIDI-neutral residual burden (Supplementary Table S9).

### 3.4. Income Reachability and Secondary Burden Signal

Income-stratified first-stage reachability disparities were stable across validation cohorts (Table 3, Panel B). Under the consistently applied primary unweighted analytic-cohort estimand, reachability showed an approximately four-fold gradient between the lowest and highest income categories, with a descriptive 2022 baseline linear probability model slope of +0.038 per income category. Because these first-stage differences occurred before conditional cost analysis, Oaxaca–Blinder residuals should be interpreted as selected-subset cost estimates among reachable adults rather than as full-population equity deficits. Survey-weighted income-stratum reachability sensitivities are reported separately in Supplementary Table S6.

Among reachable adults, income-axis Oaxaca–Blinder residual point estimates remained positive but attenuated by 58% from the 2022 holdout cohort to the 2024 transfer cohort. The fixed-pipeline weighted row-bootstrap interval excluded zero in the 2022 holdout and crossed zero marginally in the 2024 transfer, but neither interval is fully design-based because row resampling did not preserve strata or PSU structure. Therefore, the more stable income-axis finding was the strong first-stage reachability gradient, whereas the conditional income-cost signal should be interpreted cautiously. Per-covariate decomposition identified BMI as the largest structural contributor (+0.252) to the 2022 income contrast (Supplementary Table S12). IPW and mediation-relevant diagnostics are reported in the Supplement. The cross-cycle SIDI and income residuals, together with the income reachability gradient, are summarized in Supplementary Figure S1.

### 3.5. Neutral-Cost Anatomy and Multi-Axis Explorations

**Figure 4:**
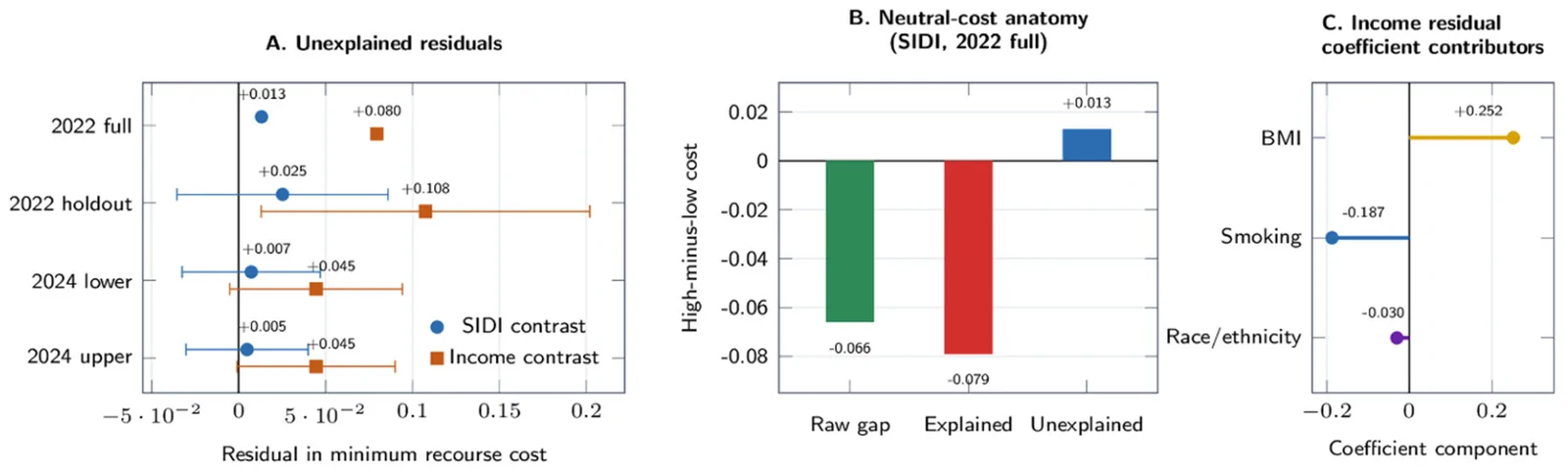
Oaxaca–Blinder residuals, neutral-cost anatomy, and income-residual coefficient contributors. Panel (**A**) shows unexplained residuals in minimum recourse cost across SIDI, and income contrasts in the 2022 full artifact, 2022 holdout, and 2024 frozen transfer analyses. The 2024 lower and upper labels refer to lower- and upper-bound missing-component coding, not low- and high-SIDI populations. Where shown, error bars in Panel (**A**) are 95% fixed-pipeline weighted individual-row bootstrap intervals and are not fully design-based BRFSS confidence intervals. Under the prespecified primary SIDI-neutral additive-cost specification, SIDI residuals were small and near-null across validation stages; alternative burden metrics were direction-dependent. Income residuals were larger in 2022 and attenuated in 2024. Panel (**B**) decomposes the 2022 full-artifact SIDI-neutral high-minus-low cost gap, showing a negative raw gap (−0.066), a larger negative explained component (−0.079), and a small positive unexplained residual (+0.013). Panel (**C**) shows coefficient contributors to the income residual, with BMI contributing positively (+0.252), smoking contributing negatively (−0.187), and race/ethnicity contributing minimally (−0.030). OB indicates Oaxaca–Blinder; SIDI, Social Indicators of Disparity Index. In Panel (**A**), blue indicates the SIDI contrast and orange indicates the income contrast. In Panel (**B**), green, red, and blue indicate the raw gap, explained component, and unexplained component, respectively. In Panel (**C**), yellow, blue, and purple indicate BMI, smoking, and race/ethnicity, respectively.

In the 2022 baseline artifact, which included 17,766 reachable adults, the higher-SIDI group had a lower mean neutral cost than the lower-SIDI group (1.116 vs. 1.182). Decomposition attributed this negative raw gap (−0.066) mainly to the explained component (−0.079), driven by greater modifiable BMI and smoking headroom, rather than to the small positive residual (+0.013) (Figure 4). Alternative burden summaries showed that this interpretation was metric-dependent: although the neutral cost metric favored higher-SIDI adults, they required slightly higher discrete action counts to cross the threshold (Supplementary Table S11). Compact robustness summaries are provided in Supplementary Table S13.

Exploratory multi-axis scans further supported axis-specificity. Income showed the largest secondary residual signal, whereas race/ethnicity and sex were near-null after conditioning on clinical covariates (Supplementary Table S14). SIDI component-level checks were also near-null (Supplementary Table S10). Within the EBM additive risk surface, BMI emerged as the largest coefficient-structure contributor (+0.252) to the income contrast (Supplementary Table S12; Figure 4). Dose–response localization relative to income category 6 showed that the unexplained residual peaked in income categories 1 (+0.098) and 2 (+0.097), attenuated in category 3 (+0.048), and was essentially absent in categories 4 and 5 (Supplementary Table S15).

Comprehensive threshold and additional robustness summaries are provided in Supplementary Tables S5 and S14. To reduce alpha inflation across these secondary contrasts, signals were prioritized using a Benjamini–Hochberg false discovery rate guardrail (*q* = 0.05) [32]. This procedure supported robustness screening without altering the primary cross-cycle SIDI-neutral estimand.

### 3.6. Staged Constraints, Pathway Mix, and Stratum-Specific Recourse Cost Profile

Staged constraint diagnostics are reported in Supplementary Table S16 to attribute how each constraint class affected candidate recourse generation. These diagnostics are not directly comparable with the primary 10.61% full-cohort reachability estimate because Supplementary Table S16 uses a 10,000-person high-risk subsample and sequentially relaxes constraints for diagnostic attribution. In contrast, the primary analysis used the full high-risk cohort, the locked adverse-risk threshold, the prespecified action ontology, exact candidate-action generation, sparsity restriction, and minimum-cost optimization. Primary-artifact action-space compatibility sensitivity across BMI-only, smoking-only, and combined sparse action sets is shown in Supplementary Figure S2 and reported in Supplementary Table S17.

The SSRCP is an implementation-oriented reporting template for institutional oversight rather than a separate statistical test. It summarizes subgroup counts, discrimination and calibration, subgroup calibration, high-risk prevalence, reachability, conditional cost, pathway composition, contrast-level residuals, uncertainty, missingness caveats, primary-artifact action-space compatibility sensitivity (Supplementary Table S17), and positive-control behavior (Supplementary Table S18). The purpose of the SSRCP is to keep predictive performance, subgroup reliability, and recourse-audit quantities separate before any recourse-enabled workflow is considered for local validation (Table 4).

## 4. Discussion

This pre-deployment recourse-governance audit produced three main findings under same-source temporal transport. First, the frozen 2022 EBM maintained stable discrimination across the 2022 full artifact, 2022 holdout, and 2024 BRFSS transfer cohorts, with survey-weighted AUCs of 0.858, 0.855, and 0.858, respectively, and preserved calibration in 2024 (slope: 1.014). Second, constrained reachability was stable but low: primary unweighted analytic-cohort estimates were 10.61%, 10.54%, and 9.82%, and corresponding survey-weighted domain estimates were 12.69%, 12.31%, and 11.30%. Third, under the prespecified primary SIDI-neutral additive-cost specification, residual conditional cost differences across SIDI strata remained small; alternative burden metrics were direction-dependent. Income showed a strong first-stage reachability gradient and a directionally positive second-stage residual in 2022 that attenuated in 2024. Positive-control simulations further showed that the reporting framework responded as intended when inequity was deliberately introduced into conditional costs or action availability.

Together, these findings show why predictive performance, reachability, conditional burden, and equity-axis contrasts should be evaluated as distinct governance quantities. A risk model may retain strong discrimination and calibration while offering no feasible candidate pathway for approximately 90% of high-risk adults under a conservative action ontology. In this setting, income was most informative for first-stage reachability, whereas SIDI-neutral conditional cost differences were bounded after conditioning on reachability. The audit therefore identifies whether deployment concern is driven primarily by a lack of feasible pathways, conditional burden among reachable adults, subgroup calibration, or axis-specific equity signals.

This study extends previous work on artificial intelligence in dentistry and health AI governance by adding a pre-deployment recourse-auditing layer to conventional predictive evaluation [15,33,34]. Conventional model evaluation focuses on discrimination, calibration, and validation, but these metrics do not determine whether high-risk classifications correspond to feasible, clinically constrained, and equitably distributed candidate actions [8,25]. To address this gap, our framework separates first-stage reachability from second-stage conditional burden, uses Oaxaca–Blinder decomposition as descriptive accounting rather than causal attribution, and evaluates temporal transport under a locked, clinically grounded action ontology.

For oral health research, the value of this framework lies in determining whether model-identified risks are associated with plausible modifiable pathways while explicitly acknowledging that existing tooth loss cannot be reversed. Irreversibility is the boundary on clinical interpretation, not a computational input that by itself caused low reachability: threshold crossing cannot restore teeth or establish treatment benefit. The available diagnostics are most consistent with limited modifiable model-space headroom under the prespecified action ontology. Only BMI, smoking status, and binge drinking were actionable. Reachable adults were younger, had higher BMI, were more frequently current smokers, and had lower predicted risk than unreachable adults, a descriptive pattern consistent with greater BMI/smoking action headroom and closer proximity to the threshold (Table 2, Panel B). The staged relaxation diagnostic showed the largest observed reduction when immutability locks were imposed, although it used a 10,000-person subsample and a different reducibility procedure and therefore cannot be read as a formal causal decomposition of the primary 10.61% estimate (Supplementary Table S16). Saved-plan compatibility reached 10.6% with BMI plus smoking and did not increase when binge drinking was added, but that analysis did not regenerate recourse under expanded action spaces (Supplementary Table S17). Accordingly, low reachability should be interpreted as a limitation of model-state actionability under the current model, threshold, constraints, and action ontology, not as an optimization failure or a claim that the clinical outcome is reversible. For unreachable individuals, automated recourse should not be surfaced under the current specification. Any future implementation as an AI-enabled medical device function would require evaluation for its intended use under applicable safety and effectiveness requirements [35]. More broadly, the need for human oversight, transparency, accountability, equity, and continuing assessment is consistent with WHO guidance on artificial intelligence for health [36].

Oaxaca–Blinder residuals should be interpreted alongside first-stage reachability because conditional cost estimates are restricted to adults who were reachable under the locked audit specification. Cost-specification transparency remains central to this interpretation [37]. SIDI-neutral and SIDI-adjusted costs answer different governance questions and should not be visually or inferentially merged. SIDI was a study-constructed, individual-level audit stratifier; it was not externally validated against geocoded deprivation indices and should not be treated as interchangeable with a validated area-deprivation measure. Income captured a distinct socioeconomic axis, with a sharper first-stage reachability gradient and a secondary conditional-burden signal. The 2024 transfer analysis attenuated the income-axis point estimate by 58% relative to the 2022 holdout cohort, from 0.108 to 0.045; the fixed-pipeline row-bootstrap interval crossed zero marginally. Whether driven by population composition shifts, row-level sampling variability, or temporal dataset shift, this attenuation highlights variation in descriptive audit quantities rather than pipeline failure [38,39].

Secondary robustness checks, including SIDI-adjusted costs, IPW analyses, and intersectional scans, supported metric dependence without altering the primary SIDI-neutral estimand. The additive EBM architecture also enabled coefficient-level localization of secondary contrasts, including the BMI contribution to the income-axis signal [17]. These outputs should therefore be interpreted as descriptive signals related to clinical profiles and governance, not as causal socioeconomic estimates. Small Oaxaca–Blinder residuals also do not guarantee uniform predictive reliability. Subgroup calibration and conditional recourse burden should remain analytically distinct. Integrating subgroup calibration directly into the SSRCP enables oversight teams to monitor whether risk scores, feasibility, and structural burden align or diverge across monitored strata.

Operationally, this framework should be used to review oral health risk prediction models before implementation and after model, threshold, or action-space updates, rather than as an autonomous clinical system. As an implementation-oriented prototype, the SSRCP should be refreshed after model updates, threshold changes, action-space revisions, or population drift. It should also be evaluated with clinical users, local workflows, interpretability requirements, and prospective monitoring plans before any recourse-enabled decision support is deployed [38]. To prevent invalid visual or inferential merging, governance dashboards should explicitly separate engineering-neutral estimates from burden-adjusted sensitivity metrics. The latter should be treated as supplemental robustness checks rather than as replacement primary estimands; detailed robustness analyses and reporting information are provided in the Supplementary Materials.

Several limitations require acknowledgment. First, BRFSS data are cross-sectional and self-reported; therefore, algorithmic recourse pathways represent computational model audits rather than observed patient adherence, treatment effectiveness, causal risk reduction, or disease reversal. This limitation is especially important for severe tooth loss, an irreversible prevalent outcome. Reducing BMI, stopping smoking, or reducing binge drinking cannot restore missing teeth, and threshold crossing should be interpreted only as movement within the fitted model feature space. Second, fixing the recourse-audit imputation, main-effects model, and threshold pipelines ensured reproducibility but omitted upstream variance from imputation, model training, threshold re-optimization, and recourse regeneration. The AUC, Brier, and Oaxaca–Blinder intervals were generated by individual-row bootstrap procedures that retained final survey weights but did not preserve BRFSS strata or PSU clustering; they are therefore fixed-pipeline weighted row-bootstrap intervals, not fully design-based BRFSS confidence intervals. The design-aware survey-domain intervals in Supplementary Table S6 apply only to weighted reachability and likewise do not propagate upstream pipeline uncertainty.

Third, the Oaxaca–Blinder decomposition was purely descriptive and conditional on the non-representative reachable subset. Despite IPW diagnostics, residual imbalances preclude causal interpretation, leaving formal selection-correction approaches, such as Heckman models, for future work [40]. Residual conditional cost differences may also reflect omitted variables, model specification, reference-group selection, measurement error, cost definition, and selection into the reachable subgroup. Fourth, SIDI was a study-constructed individual-level proxy with sparse extreme strata, particularly SIDI = 4, and lacked external validation against geocoded area-deprivation indices. Accordingly, SIDI should be interpreted only as a study-specific audit stratifier. Finally, the 2024 assessment is same-source temporal validation across BRFSS cycles, not independent external validation in a clinical population. It does not establish transportability to electronic health records, local patient case mix, clinical workflows, clinician interpretation, or live implementation settings [38,39]. External clinical and prospective local validation of the model, threshold, action space, and governance workflow would be required before any recourse-enabled decision-support use.

## 5. Conclusions

This pre-deployment audit demonstrated that stable discrimination and calibration do not necessarily translate into deployment-ready actionability. Across the 2022 full, 2022 holdout, and frozen 2024 same-source temporal-transfer cohorts, primary unweighted analytic-cohort reachability remained low (10.61%, 10.54%, and 9.82%), and corresponding survey-weighted domain sensitivities also remained limited (12.69%, 12.31%, and 11.30%). Under the prespecified primary SIDI-neutral additive-cost specification, residual conditional cost differences across SIDI strata were small, while alternative burden metrics showed direction dependence. Income-based analyses provided complementary governance information, particularly regarding first-stage reachability; the secondary conditional-cost signal was described using fixed-pipeline row-bootstrap intervals and requires cautious interpretation. The proposed framework integrates clinically constrained algorithmic recourse, two-stage assessment of reachability and conditional burden, equity-axis decomposition, cost-specification stress testing, and same-source temporal transport within an implementation-oriented Stratum-Specific Recourse Cost Profile. These outputs are descriptive model-space audit signals rather than treatment recommendations, causal risk reductions, or reversal of an existing outcome. External clinical and prospective local validation would be required before recourse-enabled use in oral health decision support.

## Figures and Tables

**Table 1:** Baseline characteristics of the 2022 high-risk recourse cohort by SIDI stratum.

Characteristic	Total High-Risk	Low SIDI (<2)	High SIDI (≥2)	High-Low
N, unweighted	167,499	149,117	18,382	
Age, mean years	64.9	66.5	54.6	−11.9
Female	53.2%	53.6%	50.5%	−3.1%
Married	45.6%	48.5%	27.2%	−21.3%
College graduate	9.8%	10.8%	3.8%	−7.0%
Income < $25,000	30.6%	26.0%	60.3%	+34.3%
Insured	93.7%	95.2%	83.5%	−11.7%
Could not see doctor due to cost	13.2%	10.7%	29.4%	+18.7%
Fair or poor general health	37.6%	34.4%	57.8%	+23.4%
BMI, mean kg/m^2^	29.4	29.2	30.5	+1.3
Current smoker	25.7%	22.8%	44.4%	+21.6%
Binge drinking	8.3%	7.8%	11.8%	+4.0%
Diabetes	27.3%	27.2%	28.4%	+1.2%
Prior heart attack	11.4%	11.4%	11.9%	+0.5%
Prior stroke	8.9%	8.5%	11.6%	+3.1%
Asthma	18.3%	17.1%	25.9%	+8.8%
Arthritis	54.1%	55.0%	48.2%	−6.8%
Cancer	16.3%	17.3%	9.8%	−7.5%
Kidney disease	8.6%	8.5%	9.3%	+0.8%
COPD	17.8%	17.1%	22.0%	+4.9%
Severe tooth loss	33.8%	33.2%	38.2%	+5.0%

Note: Values are survey-weighted percentages unless otherwise indicated; counts are unweighted. Low SIDI was defined as SIDI < 2 and high SIDI as SIDI ≥ 2. The 19 EBM predictors are summarized using clinically compact categories; severe tooth loss is shown as the cohort outcome context.

**Table 2:** Evaluation of recourse feasibility and subpopulation characterization.

**Panel A. Predictive Performance and Macro-Level Reachability Across Validation Stages**			
**Analysis**	**2022 Full Artifact**	**2022 Holdout**	**2024 Frozen Transfer**
N	433,772	86,755	448,213
High-risk N	167,499	33,537	174,664
Weighted AUC	0.858 [0.855, 0.861]	0.855 [0.847, 0.863]	0.858 [0.854, 0.861]
Weighted Brier score	0.088 [0.087, 0.089]	0.088 [0.085, 0.091]	0.086 [0.084, 0.087]
High-risk subset Brier score	0.197	0.197	0.195
Weighted calibration slope	1.010	0.999	1.014
Weighted ECE	0.007	0.006	0.006
Reachable N (%; primary unweighted analytic cohort)	17,766 (10.61%)	3536 (10.54%)	17,152 (9.82%)
Survey-weighted reachability sensitivity [95% CI]	12.69% [12.33%, 13.07%]	12.31% [11.54%, 13.12%]	11.30% [10.95%, 11.65%]
**Panel B. Clinical characteristics of reachable versus unreachable high-risk adults in the 2022 full artifact**			
**Characteristic**	**Reachable**	**Unreachable**	**Reachable-Unreachable**
High-risk adults, N	17,766	149,733	
Share of high-risk cohort	10.61%	89.39%	−78.79%
Age, mean years	49.3	67.2	−17.9
BMI, mean kg/m^2^	32.1	29.0	+3.1
Current smoker	58.3%	21.0%	+37.3%
Severe tooth loss	18.2%	36.1%	−17.9%
Predicted risk, mean	0.169	0.357	−0.189

Note: Reachable N (%) reports the primary unweighted analytic-cohort proportion. The survey-weighted reachability row reports BRFSS survey-domain estimates with design-aware 95% confidence intervals using WEIGHT, STRATA, and PSU; full specifications and subgroup estimates appear in Supplementary Table S6. Bracketed AUC and Brier values are 95% fixed-model weighted row-bootstrap intervals that did not preserve strata or PSU structure. The 2022 full artifact interval is descriptive because it includes the training split, whereas the 2022 holdout and 2024 intervals reflect validation and same-source temporal-transport performance. AUC indicates area under the receiver operating characteristic curve; ECE, expected calibration error.

**Table 3:** Multi-axis equity audits across validation phases: reachability gradients and unexplained Oaxaca–Blinder cost residuals.

**Panel A. Primary SIDI-Neutral Conditional Cost Decomposition Contrasts**			
**Analysis**	**SIDI Missing-Component Coding**	**SIDI Unexplained Residual [95% CI]**	**Income Unexplained Residual [95% CI]**
2022 baseline artifact	Original	0.013	0.080
2022 holdout	Lower-bound	0.025 [−0.035, 0.086]	0.108 [0.013, 0.202]
2024 frozen transfer	Lower-bound	0.007 [−0.026, 0.047]	0.045 [−0.001, 0.095]
2024 frozen transfer	Upper-bound	0.005 [−0.031, 0.040]	0.045 [−0.003, 0.090]
**Panel B. Income-stratified first-stage reachability gradients and secondary conditional residuals**			
**Analysis**	**Lowest-income reachability**	**Highest-income reachability**	**Income unexplained residual**
2022 baseline artifact	5.8%	26.9%	0.080
2022 holdout	6.6%	27.0%	0.108
2024 frozen transfer	5.6%	24.2%	0.045

Note: The Oaxaca–Blinder outcome was minimum recourse cost under the primary SIDI-neutral additive-cost specification. The SIDI contrast compares SIDI ≥ 2 versus SIDI 0–1; the Oaxaca–Blinder income contrast compares income categories 1–2 versus 5–6, whereas the Panel B reachability columns show the extreme individual categories 1 and 6. Reachability percentages are primary unweighted high-risk analytic-cohort proportions; the 2022 baseline income-category values were corrected to 5.8% and 26.9% so the estimand is consistent across all stages. Oaxaca–Blinder point estimates used survey-weighted least squares among reachable adults. Their confidence intervals were derived from 1000 fixed-pipeline weighted individual-row bootstrap replicates that did not preserve STRATA or PSU structure and are not fully design-based. Survey-weighted reachability sensitivities are reported separately in Supplementary Table S6.

**Table 4:** Stratum-Specific Recourse Cost Profile for governance reporting.

Audit Measure	Reference Estimate	Monitored Estimate	Contrast/Uncertainty	Governance Interpretation
High-risk N, 2022 full artifact	Low SIDI: 149,117	High SIDI: 18,382	Descriptive counts	Size of monitored high-risk strata
Reachable N, 2022 full artifact	Low SIDI: 15,234	High SIDI: 2532	Descriptive counts	Adults for whom sparse candidate recourse could be surfaced
Mean SIDI-neutral cost, 2022 full artifact	1.182	1.116	High-minus-low raw gap: −0.066	Neutral model-movement burden among reachable adults
Weighted Brier score, 2022 high-risk subset	0.196	0.208	Difference: +0.012	Prediction error reported separately from recourse burden
Expected calibration error, 2022 high-risk subset	0.014	0.035	Difference: +0.021	Subgroup calibration reported separately from recourse burden
Calibration slope, 2022 high-risk subset	0.913	0.798	Difference: −0.115	Higher-SIDI stratum showed poorer probability reliability
Primary SIDI-neutral Oaxaca–Blinder residual	Not a stratum value	Not a stratum value	+0.013; contrast-level statistic	Bounded residual conditional cost difference; monitor rather than interpret causally
2024 temporal transport check	Same locked pipeline	Lower-/upper-bound SIDI coding	SIDI residuals: 0.007/0.005	Same-source transfer did not amplify SIDI conditional burden
Dominant pathway types	BMI, smoking	Smoking, BMI	Pathway mix	Candidate pathway composition should be reviewed before local use
Recommended audit action	Routine monitoring	Enhanced monitoring	Governance action, not clinical prescription	Recompute after model, threshold, action-space, or population drift

Note: The SSRCP is a governance dashboard, not a separate statistical test. The Oaxaca–Blinder residual is a contrast-level statistic and should not be interpreted as a per-stratum value. SIDI indicates Social Indicators of Disparity Index.

## Data Availability

The BRFSS 2022 and 2024 public-use datasets and documentation are available from the Centers for Disease Control and Prevention (CDC) at https://www.cdc.gov/brfss (accessed on 27 August 2026). Study-specific derived analytic files, trained models, recourse outputs, and analysis code have been archived on Zenodo at https://doi.org/10.5281/zenodo.20783950.

## Supplementary Materials

The following supporting information can be downloaded at: https://www.mdpi.com/article/10.3390/bioengineering13091020/s1. Table S1. Explainable Boosting Machine model settings. Table S2. Construction of the Social Indicators of Disparity Index. Table S3. Distribution of Social Indicators of Disparity Index scores across analytic cohorts. Table S4. Predictor roles, action ontology, time horizon, and recourse handling. Table S5. Threshold sensitivity, BRFSS survey-weighted reachability, and inverse-probability-weighted supplemental diagnostics. Table S6. Primary unweighted analytic-cohort reachability and BRFSS survey-weighted domain sensitivity with design-aware confidence intervals. Table S7. Oaxaca-Blinder decomposition and bootstrap uncertainty specification. Table S8. Robustness of SIDI-neutral Oaxaca-Blinder decomposition to missing-component coding. Table S9. Alternative SIDI cutpoint sensitivity using SIDI-neutral recourse cost. Table S10. Component-level Oaxaca-Blinder decomposition for individual SIDI indicators. Table S11. Alternative burden metrics among reachable adults. Table S12. Per-covariate Oaxaca-Blinder decomposition for income categories 1–2 versus 5–6. Table S13. Additional robustness summaries. Table S14. Exploratory 2022 multi-axis Oaxaca-Blinder decomposition using SIDI-neutral cost. Table S15. Income dose–response Oaxaca-Blinder decomposition using income category 6 as the reference group. Table S16. Constraint-relaxation diagnostic based on a 10,000-person high-risk subsample. Table S17. Primary-artifact action-space compatibility sensitivity. Table S18. Positive-control simulations for deliberately injected prescriptive inequity. Figure S1. Cross-cycle equity-axis comparison of SIDI residuals, income residuals, and income reachability gradients. Panel A shows SIDI Oaxaca–Blinder residuals under the prespecified primary SIDI-neutral additive-cost specification; the 2024 lower- and upper-bound labels refer to missing-component coding, not to low- and high-SIDI populations. Panel B shows income residuals with fixed-pipeline weighted row-bootstrap intervals; those intervals did not preserve STRATA or PSU structure. Panel C consistently applies the primary unweighted analytic-cohort reachability estimand across stages. The verified 2022 baseline values are 5.8% for income category 1 and 26.9% for category 6; the holdout values are 6.6% and 27.0%, and the 2024 values are 5.6% and 24.2%. Survey-weighted income-stratum sensitivities are reported in Supplementary Table S6. SIDI and income are distinct audit axes. OB indicates Oaxaca–Blinder; SIDI, Social Indicators of Disparity Index; PSU, primary sampling unit. Figure S2. Primary-artifact saved-plan compatibility sensitivity in the 2022 full artifact. Compatible reachability uses unweighted analytic denominators. BMI-only and smoking-only saved-plan action spaces yielded 5.1% and 4.8% compatibility. Combining BMI and smoking yielded 9.9% when limited to one action and 10.6% when allowing two actions. Adding binge drinking under k ≤ 3 did not increase compatibility beyond 10.6%. This diagnostic classifies the actions present in saved optimal plans and does not regenerate or re-optimize recourse under expanded action spaces.
