# Supplementary material for: Pre-Deployment Audit of Actionability and Equity in Severe Tooth Loss Prediction Using Constrained Algorithmic Recourse and Temporal Validation
Source: Bioengineering (Basel). 2026 Sep 1;13(9):1020. doi: 10.3390/bioengineering13091020 (PMC13603997; doi:10.3390/bioengineering13091020)
Supplement: Supplementary file 1 [file bioengineering-13-01020-s001.zip › bioengineering-4517342-supplementary.pdf]

SUPPLEMENTARY

Supplementary Table S1. Explainable Boosting Machine model settings.

Setting	Value
Model class	Explainable Boosting Classifier
Number of predictors	19
Number of terms	19
Interaction terms	0
Maximum bins	1024
Learning rate	0.01
Maximum rounds	25,000
Minimum samples per leaf	2
Outer bags	14
Inner bags	0
Validation fraction	0.15
Early stopping rounds	50
Early stopping tolerance	0.0
Smoothing rounds	200
Random seed	42
Prespecified adverse-risk threshold	$\tau = 0.1295$ (0.130 rounded)

Note: The EBM was specified as a 19-feature, main-effects-only audit artifact. Interaction terms were disabled to preserve additive feature-level accounting for the recourse audit. The adverse-risk threshold was prespecified on the 2022 training data and locked before holdout and 2024 temporal-transfer analyses.

Supplementary Table S2. Construction of the Social Indicators of Disparity Index.

Component	Social determinants of health domain	BRFSS variable	Deprivation indicator definition	SIDI contribution
Housing instability	Neighborhood and built environment	RENTHOM1	Renting rather than owning the current residence	1 if present; 0 otherwise
Employment vulnerability	Economic stability	EMPLOY1	Out of work for 1 year or more, or unable to work	1 if present; 0 otherwise
Delayed routine care	Health care access and quality	CHECKUP1	Last routine checkup 2 or more years ago, or never	1 if present; 0 otherwise
Household competing demands	Social and community context	HHADULT	Four or more adults in the household	1 if present; 0 otherwise

Note: SIDI was calculated as the unweighted sum of four binary deprivation indicators, yielding a score from 0 to 4. Higher scores indicate greater accumulated deprivation across the study-defined individual-level audit domains. SIDI was used as a prespecified audit stratifier, not as a newly validated deprivation index or a substitute for geocoded area-level deprivation measures.

Supplementary Table S3. Distribution of Social Indicators of Disparity Index scores across analytic cohorts.

Dataset/coding rule	SIDI 0	SIDI 1	SIDI 2	SIDI 3	SIDI 4	Total
2022 analytic cohort, lower-bound coding	259,960	129,165	39,642	4,817	188	433,772
2024 analytic cohort, lower-bound coding	266,150	133,984	42,489	5,399	191	448,213
2024 analytic cohort, upper-bound coding	200,598	185,457	54,177	7,690	291	448,213

Note: Values are unweighted analytical counts. SIDI was calculated as the sum of four binary deprivation indicators and ranged from 0 to 4. Lower-bound coding treated missing SIDI components as absent unless deprivation was observed. Upper-bound coding treated missing SIDI components as present to evaluate the sensitivity of 2024 transfer results to missing-component assumptions. SIDI indicates Social Indicators of Disparity Index.

Supplementary Table S4. Predictor roles, action ontology, time horizon, and recourse handling.

Predictor	EBM role	Recourse actionability	Candidate perturbation or handling	Cost weight	OB covariate	Expected time horizon	Directionality/reversibility	Primary actor locus	Clinical interpretation	Causal benefit assumed?
Age	Predictor	Immutable	Held fixed	Not applicable	Yes	Not actionable	Held fixed	Not patient-actionable	Audit constraint preventing invalid recommendations	No
Sex	Predictor	Immutable	Held fixed	Not applicable	Yes	Not actionable	Held fixed	Not patient-actionable	Audit constraint preventing invalid recommendations	No
Marital status	Predictor	Immutable	Held fixed	Not applicable	No	Not actionable	Held fixed	Not patient-actionable	Audit constraint preventing invalid recommendations	No
Education	Predictor	Immutable	Held fixed	Not applicable	No	Not actionable	Held fixed	Not patient-actionable	Audit constraint preventing invalid recommendations	No
Income	Predictor	Immutable	Held fixed	Not applicable	No	Not actionable	Held fixed	Not patient-actionable	Audit constraint preventing invalid recommendations	No
Insurance	Predictor	Held fixed	Not optimized in the current recourse engine	Not applicable	No	Not optimized in current audit	Held fixed	May require clinical or system-level intervention if used in future	Excluded from the current recourse action space	No
Cost barrier	Predictor	Held fixed	Not optimized; direct cost-related access was excluded from SIDI to avoid overlap	Not applicable	No	Not optimized in current audit	Held fixed	May require clinical or system-level intervention if used in future	Excluded from the current recourse action space	No

Predictor	EBM role	Recourse actionability	Candidate perturbation or handling	Cost weight	OB covariate	Expected time horizon	Directionality/reversibility	Primary actor locus	Clinical interpretation	Causal benefit assumed?
General health	Predictor	Held fixed	Not optimized as a direct prescriptive action	Not applicable	No	Not optimized in current audit	Held fixed	May require clinical or system-level intervention if used in future	Excluded from the current recourse action space	No
BMI	Predictor; modifiable recourse feature	Modifiable with safety constraints	Decreases allowed only when BMI > 25 kg/m²; never below 18.5 kg/m²; minimum change 1.0 kg/m²; candidates generated from EBM bin midpoints and filtered by monotone risk-score improvement	0.7052	Yes	Months to years	Decrease only when BMI > 25 kg/m²; not below 18.5 kg/m²; partially reversible	Patient plus health-system support	Hypothetical model-state movement in BMI, not verified weight-loss effectiveness	No
Smoking status	Predictor; modifiable recourse feature	Directionally modifiable	Current daily or some-day smoker can move to former-smoker state only	0.7428	Yes	Months to years	Current smoker to former smoker only; never-smoker state not allowed	Patient plus cessation support	Hypothetical cessation-state movement in the model	No

Predictor	EBM role	Recourse actionability	Candidate perturbation or handling	Cost weight	OB covariate	Expected time horizon	Directionality/reversibility	Primary actor locus	Clinical interpretation	Causal benefit assumed?
Binge drinking	Predictor; modifiable recourse feature	Directionally modifiable	Binge drinker can move to non-binge drinker state	0.1520	No	Weeks to months	Binge to non-binge only; behavior may relapse	Patient plus behavioral support	Hypothetical model-state movement for alcohol-risk behavior	No
Diabetes	Predictor	Held fixed	Not optimized; BRFSS diagnostic status is not a disease-management action	Not applicable	No	Not optimized in current audit	Held fixed	May require clinical or system-level intervention if used in future	Excluded from the current recourse action space	No
Heart attack	Predictor	Held fixed	Not optimized; diagnostic history/status variable	Not applicable	No	Not optimized in current audit	Held fixed	May require clinical or system-level intervention if used in future	Excluded from the current recourse action space	No
Stroke	Predictor	Held fixed	Not optimized; diagnostic history/status variable	Not applicable	No	Not optimized in current audit	Held fixed	May require clinical or system-level intervention if used in future	Excluded from the current recourse action space	No
Asthma	Predictor	Held fixed	Not optimized; diagnostic history/status variable	Not applicable	No	Not optimized in current audit	Held fixed	May require clinical or system-level intervention if used in	Excluded from the current recourse action space	No

Predictor	EBM role	Recourse actionability	Candidate perturbation or handling	Cost weight	OB covariate	Expected time horizon	Directionality/reversibility	Primary actor locus	Clinical interpretation	Causal benefit assumed?
								future		
Arthritis	Predictor	Held fixed	Not optimized; diagnostic history/status variable	Not applicable	No	Not optimized in current audit	Held fixed	May require clinical or system-level intervention if used in future	Excluded from the current recourse action space	No
Cancer	Predictor	Held fixed	Not optimized; diagnostic history/status variable	Not applicable	No	Not optimized in current audit	Held fixed	May require clinical or system-level intervention if used in future	Excluded from the current recourse action space	No
Kidney disease	Predictor	Held fixed	Not optimized; diagnostic history/status variable	Not applicable	No	Not optimized in current audit	Held fixed	May require clinical or system-level intervention if used in future	Excluded from the current recourse action space	No
COPD	Predictor	Held fixed	Not optimized; diagnostic history/status variable	Not applicable	No	Not optimized in current audit	Held fixed	May require clinical or system-level intervention if used in future	Excluded from the current recourse action space	No

Note: EBM indicates Explainable Boosting Machine; OB, Oaxaca-Blinder. “Held fixed” indicates that the feature was not modified during candidate recourse generation. “Immutable” denotes variables that were locked because changing them would be clinically or ethically invalid. Modifiable recourse features were limited to BMI, smoking status, and binge drinking under the prespecified action ontology. All recourse outputs represent hypothetical model-state movement

and should not be interpreted as treatment recommendations, causal risk reduction, or reversal of severe tooth loss.

Supplementary Table S5. Threshold sensitivity, BRFSS survey-weighted reachability, and inverse-probability-weighted supplemental diagnostics.

Diagnostic	Setting/contrast	Key estimate	Confidence interval/balance summary	Interpretation
Threshold sensitivity	Youden index	Threshold = 0.1295	High-risk N = 167,499; reachable N = 17,766; raw unreachability = 89.4%	Primary operating point used in the manuscript.
Threshold sensitivity	Youden index -10%	Threshold = 0.1166	High-risk N = 179,286; reachable N = 9,096; raw unreachability = 94.9%	Lowering the threshold expanded the high-risk pool but further reduced reachability.
Threshold sensitivity	Youden index +10%	Threshold = 0.1425	High-risk N = 156,746; reachable N = 11,801; raw unreachability = 92.5%	Raising the threshold preserved the qualitative pattern of low reachability.
Threshold sensitivity	Fixed sensitivity of 80%	Threshold = 0.1461	High-risk N = 153,908; reachable N = 10,744; raw unreachability = 93.0%	This alternative operating point still left most high-risk adults unreachable.
IPW comparison	2022 income categories 1-2 vs. 5-6, baseline	Raw gap = +0.0521; explained component = -0.0274; unexplained residual = +0.0795	95% CI +0.0348 to +0.1272	A positive income-axis residual was observed in the reachable subset before IPW reweighting.
IPW comparison	2022 income categories 1-2 vs. 5-6, stabilized IPW	Raw gap = +0.0063; explained component = -0.0333; unexplained residual = +0.0396	95% CI +0.0028 to +0.0756	The residual direction was preserved but attenuated after IPW reweighting.
BRFSS survey-weighted reachability	2024 lower-bound SIDI coding	Survey-weighted reachable = 11.30%	95% design-aware CI 10.95% to 11.65%	Population-oriented BRFSS survey-weight sensitivity; this estimate does not use inverse-probability weighting.
BRFSS survey-weighted reachability	2024 upper-bound SIDI coding	Survey-weighted reachable = 11.30%	95% design-aware CI 10.95% to 11.65%	Reachability is unchanged by the alternative SIDI missing-component coding; the full design specification appears in Table S6.

Diagnostic	Setting/contrast	Key estimate	Confidence interval/balance summary	Interpretation
IPW conditional-cost/balance diagnostic	2024 lower-bound SIDI coding	IPW-adjusted mean reachable cost = 1.4756	Effective sample size = 9,346; maximum absolute SMD after IPW: SIDI = 0.069, income = 0.211	SIDI balance improved after inverse-probability weighting, although some income imbalance remained.
IPW conditional-cost/balance diagnostic	2024 upper-bound SIDI coding	IPW-adjusted mean reachable cost = 1.5143	Effective sample size = 9,877; maximum absolute SMD after IPW: SIDI = 0.037, income = 0.213	The same balance pattern was observed under upper-bound SIDI coding.

Note: Threshold sensitivity analyses evaluated whether low reachability depended on the prespecified adverse-risk threshold. BRFSS survey-weighted reachability uses the final sampling weight and is distinct from inverse-probability weighting. IPW analyses were supplemental selection/balance diagnostics for conditional cost and were not used to redefine the primary unweighted reachability or primary SIDI-neutral estimand. Full design-aware weighted reachability results appear in Supplementary Table S6. IPW indicates inverse-probability weighting; SIDI, Social Indicators of Disparity Index; SMD, standardized mean difference; CI, confidence interval.

Supplementary Table S6. Primary unweighted analytic-cohort reachability and BRFSS survey-weighted domain sensitivity with design-aware confidence intervals.

Stage	Subgroup	High-risk N	Reachable N	Primary unweighted %	Survey-weighted % [95% design CI]
2022 full (N=433,772)	Overall	167,499	17,766	10.61%	12.69% [12.33%, 13.07%]
2022 full (N=433,772)	Low SIDI (0-1)	149,117	15,234	10.22%	12.20% [11.81%, 12.59%]
2022 full (N=433,772)	High SIDI (2-4)	18,382	2,532	13.77%	15.93% [14.85%, 17.07%]
2022 full (N=433,772)	Income 1-2	46,024	2,527	5.49%	7.23% [6.72%, 7.76%]
2022 full (N=433,772)	Income 5-6	45,177	8,401	18.60%	21.55% [20.69%, 22.44%]
2022 full (N=433,772)	Income 1	15,586	905	5.81%	7.29% [6.49%, 8.19%]
2022 full (N=433,772)	Income 2	30,438	1,622	5.33%	7.19% [6.56%, 7.87%]
2022 full (N=433,772)	Income 3	38,946	3,028	7.77%	10.74% [10.00%, 11.53%]
2022 full (N=433,772)	Income 4	37,352	3,810	10.20%	12.32% [11.58%, 13.10%]

Stage	Subgroup	High-risk N	Reachable N	Primary unweighted %	Survey-weighted % [95% design CI]
2022 full (N=433,772)	Income 5	38,115	6,498	17.05%	19.62% [18.73%, 20.55%]
2022 full (N=433,772)	Income 6	7,062	1,903	26.95%	30.26% [27.84%, 32.79%]
2022 holdout (N=86,755)	Overall	33,537	3,536	10.54%	12.31% [11.54%, 13.12%]
2022 holdout (N=86,755)	Low SIDI (0-1)	29,900	3,031	10.14%	12.02% [11.19%, 12.90%]
2022 holdout (N=86,755)	High SIDI (2-4)	3,637	505	13.89%	14.14% [12.28%, 16.23%]
2022 holdout (N=86,755)	Income 1-2	9,228	528	5.72%	7.51% [6.43%, 8.74%]
2022 holdout (N=86,755)	Income 5-6	9,123	1,649	18.08%	20.66% [18.89%, 22.56%]
2022 holdout (N=86,755)	Income 1	3,129	205	6.55%	9.13% [7.09%, 11.68%]
2022 holdout (N=86,755)	Income 2	6,099	323	5.30%	6.59% [5.45%, 7.96%]
2022 holdout (N=86,755)	Income 3	7,726	604	7.82%	10.40% [8.88%, 12.14%]

Stage	Subgroup	High-risk N	Reachable N	Primary unweighted %	Survey-weighted % [95% design CI]
2022 holdout (N=86,755)	Income 4	7,460	755	10.12%	11.45% [9.92%, 13.18%]
2022 holdout (N=86,755)	Income 5	7,656	1,253	16.37%	18.42% [16.68%, 20.30%]
2022 holdout (N=86,755)	Income 6	1,467	396	26.99%	31.27% [25.96%, 37.13%]
2024 transfer (N=448,213)	Overall	174,664	17,152	9.82%	11.30% [10.95%, 11.65%]
2024 transfer (N=448,213)	Low SIDI (0-1)	155,379	14,731	9.48%	10.85% [10.49%, 11.23%]
2024 transfer (N=448,213)	High SIDI (2-4)	19,285	2,421	12.55%	14.30% [13.26%, 15.40%]
2024 transfer (N=448,213)	Income 1-2	42,458	2,176	5.13%	6.98% [6.40%, 7.61%]
2024 transfer (N=448,213)	Income 5-6	54,417	8,794	16.16%	17.80% [17.10%, 18.52%]
2024 transfer (N=448,213)	Income 1	14,265	799	5.60%	7.61% [6.53%, 8.85%]
2024 transfer (N=448,213)	Income 2	28,193	1,377	4.88%	6.65% [5.99%, 7.38%]

Stage	Subgroup	High-risk N	Reachable N	Primary unweighted %	Survey-weighted % [95% design CI]
2024 transfer (N=448,213)	Income 3	38,337	2,509	6.54%	8.14% [7.50%, 8.83%]
2024 transfer (N=448,213)	Income 4	39,452	3,673	9.31%	11.13% [10.35%, 11.95%]
2024 transfer (N=448,213)	Income 5	45,104	6,538	14.50%	16.21% [15.47%, 16.98%]
2024 transfer (N=448,213)	Income 6	9,313	2,256	24.22%	24.71% [22.90%, 26.60%]

Note: Primary reachability is the unweighted proportion among high-risk analytic records. The survey-weighted sensitivity is a Taylor-linearized domain ratio estimated from the full analytic cohort with the high-risk indicator defining the domain and with BRFSS WEIGHT, STRATA, and PSU. Logit-transformed t confidence intervals used centered singleton-PSU adjustment as the primary rule. Certainty-PSU and average-variance sensitivity rules produced a maximum absolute endpoint difference of 0.27 percentage points across all reported intervals. The 2022 holdout interval is conditional on the fixed partition. These intervals condition on the completed analytic data, frozen EBM, threshold, and recourse outputs and do not propagate imputation, model-training, threshold-selection, or recourse-generation uncertainty. SIDI indicates Social Indicators of Disparity Index; CI, confidence interval; PSU, primary sampling unit.

Supplementary Table S7. Oaxaca-Blinder decomposition and bootstrap uncertainty specification.

Item	Specification
Analysis cohort	Reachable high-risk adults only
Primary contrast	High SIDI, defined as SIDI 2-4, minus low SIDI, defined as SIDI 0-1
Outcome	SIDI-neutral normalized additive recourse cost
Main Oaxaca-Blinder covariates	Age, sex, BMI, smoking status, and race/ethnicity
Regression estimator	Weighted least squares estimated separately within low-SIDI and high-SIDI groups
Main Table 3 uncertainty	1,000 nonparametric case-resampling bootstrap replicates; percentile 95% confidence interval; random seed = 42
Exploratory 2022 uncertainty	500 weighted case-resampling bootstrap replicates; percentile 95% confidence interval; random seed = 42
Resampling unit	Individual rows from the reachable cohort, sampled with replacement
Components held fixed during bootstrap	Completed analytic dataset, EBM, adverse-risk threshold, high-risk classification, and recourse outputs
Uncertainty target	Sampling variation in the final reachable-cohort Oaxaca-Blinder decomposition, conditional on the fixed upstream pipeline
Excluded uncertainty sources	Imputation uncertainty, model-training uncertainty, threshold-selection uncertainty, and recourse-generation uncertainty
Survey-design structure in resampling	Final BRFSS survey weights were retained in weighted estimators, but individual-row resampling did not preserve STRATA or PSU clustering.
Interval interpretation	Fixed-pipeline weighted row-bootstrap intervals; not fully design-based BRFSS confidence intervals.

Note: The bootstrap procedure quantified individual-row sampling variation in the final decomposition stage only. Final survey weights were retained in the weighted least-squares estimators, but STRATA and PSU clustering were not preserved; consequently, these are fixed-pipeline weighted row-bootstrap intervals rather than fully design-based BRFSS confidence intervals. The intervals also do not propagate uncertainty from imputation, model fitting, threshold selection, or candidate recourse generation. SIDI indicates Social Indicators of Disparity Index; EBM, Explainable Boosting Machine; PSU, primary sampling unit.

Supplementary Table S8. Robustness of SIDI-neutral Oaxaca-Blinder decomposition to missing-component coding.

Analysis	SIDI raw gap	SIDI explained component	SIDI unexplained residual	Interpretation
2022 full saved artifact	-0.0661	-0.0793	+0.0132	Baseline SIDI-neutral residual was small.
2022 holdout neutral analysis	-0.0886	-0.1139	+0.0253	Holdout residual remained small; the confidence interval crossed zero in the main analysis.
2024 frozen transfer, lower-bound coding	-0.0811	-0.0884	+0.0073	Residual was near-null under conservative missing-component coding.
2024 frozen transfer, upper-bound coding	-0.0733	-0.0782	+0.0049	Residual was near-null under upper-bound missing-component coding.

Note: The SIDI raw gap represents the high-SIDI minus low-SIDI difference in mean SIDI-neutral normalized recourse cost among reachable adults. The explained component reflects the portion associated with included Oaxaca-Blinder covariates, whereas the unexplained residual represents the remaining conditional cost difference not accounted for by those covariates. Lower-bound coding treated missing SIDI components as absent unless deprivation was observed; upper-bound coding treated missing components as present. SIDI indicates Social Indicators of Disparity Index.

Supplementary Table S9. Alternative SIDI cutpoint sensitivity using SIDI-neutral recourse cost.

Cutpoint contrast	Reference N	Disadvantaged N	Raw gap	Explained component	Unexplained residual [95% CI]	Interpretation
SIDI 0 vs. SIDI ≥ 1	9,422	8,344	-0.1140	-0.1050	-0.009 [-0.039 to +0.022]	The any-deprivation cutpoint produced a near-null unexplained residual.
SIDI 0-1 vs. SIDI ≥ 2	15,234	2,532	-0.0661	-0.0800	+0.013 [-0.022 to +0.055]	Primary manuscript contrast; residual remained bounded under the neutral cost specification.
SIDI 0-2 vs. SIDI ≥ 3	17,418	348	-0.1566	-0.0710	-0.0857 [-0.133 to -0.033]	Exploratory contrast only because the high-SIDI group was sparse.

Note: The outcome was SIDI-neutral normalized recourse cost among reachable adults. Raw gaps are calculated as disadvantaged minus reference group differences. The explained component reflects included Oaxaca-Blinder covariates, whereas the unexplained residual represents the remaining conditional cost difference not accounted for by those covariates. The SIDI 0-1 versus SIDI ≥ 2 contrast was the prespecified primary contrast used in the manuscript. SIDI indicates Social Indicators of Disparity Index.

Supplementary Table S10. Component-level Oaxaca-Blinder decomposition for individual SIDI indicators.

SIDI component	Reference N	Disadvantaged N	Raw gap	Unexplained residual [95% CI]	One-sided bootstrap *P* value/interpretation
Housing instability	12,609	5,157	-0.068	+0.008 [-0.022 to +0.036]	*P* = .280; near-null residual
Employment vulnerability	15,950	1,816	+0.042	+0.012 [-0.021 to +0.048]	*P* = .256; near-null residual
Delayed routine care	15,133	2,633	-0.208	-0.031 [-0.067 to +0.009]	*P* = .936; near-null residual
Large household	16,135	1,631	-0.010	-0.001 [-0.038 to +0.045]	*P* = .528; near-null residual

Note: Component-level analyses decomposed SIDI-neutral normalized recourse cost among reachable adults according to each individual SIDI indicator. Raw gaps are calculated as disadvantaged minus reference group differences. The unexplained residual represents the remaining conditional cost difference not accounted for by included Oaxaca-Blinder covariates. These analyses were interpreted as exploratory robustness checks for the SIDI audit stratifier, not as separate component-level causal estimates. SIDI indicates Social Indicators of Disparity Index; CI, confidence interval.

Supplementary Table S11. Alternative burden metrics among reachable adults.

Burden metric	Low-SIDI mean	High-SIDI mean	High-minus-low gap	Gap, %	Direction
SIDI-neutral additive cost	1.1825	1.1164	-0.0661	-5.6%	Low SIDI > High SIDI
Action count	1.0751	1.0972	+0.0221	+2.1%	High SIDI > Low SIDI
Equal-feature cost	1.0751	1.0972	+0.0221	+2.1%	High SIDI > Low SIDI
Risk reduction	0.0581	0.0673	+0.0092	+15.9%	High SIDI > Low SIDI
Cost per unit risk reduction	93.4282	59.1084	-34.3198	-36.7%	Low SIDI > High SIDI

Note: Values are calculated among reachable high-risk adults in the 2022 full artifact. The high-minus-low gap compares high-SIDI adults with low-SIDI adults. Alternative burden metrics were used to evaluate metric dependence of the recourse-audit findings. The SIDI-neutral additive cost suggested lower average model-movement burden among high-SIDI adults, whereas action count, equal-feature cost, and required risk reduction were slightly higher among high-SIDI adults. These metrics answer different audit questions and should not be interpreted as interchangeable burden measures. SIDI indicates Social Indicators of Disparity Index.

Supplementary Table S12. Per-covariate Oaxaca-Blinder decomposition for income categories 1-2 versus 5-6.

Covariate	Explained component	Unexplained coefficient-structure component
Intercept	0.000	+0.038
Age	-0.047	+0.009
Sex	-0.003	-0.001
BMI	+0.013	+0.252
Smoking status	+0.003	-0.187
Race/ethnicity	+0.006	-0.030
**Total**	**-0.027**	**+0.079**

Note: The contrast compares reachable adults in income categories 1-2 with those in income categories 5-6. The outcome was SIDI-neutral normalized recourse cost. The explained component reflects differences in covariate distributions between income groups, whereas the unexplained coefficient-structure component reflects differences in the covariate-cost association structure not accounted for by the included variables. These per-covariate terms are descriptive decomposition quantities and should not be interpreted as causal effects of BMI, smoking status, or any other covariate.

Supplementary Table S13. Additional robustness summaries.

Diagnostic	Estimate/interval	Interpretation
Design-effect sensitivity interval for raw cost gap	-0.099 to -0.033	The raw cost-gap direction remained stable under conservative design-effect sensitivity.
Alternative SIDI cutpoints, raw-gap range	-0.157 to -0.114	Alternative SIDI binarizations preserved the raw-gap direction.
Action-count raw gap	+0.022	High-SIDI adults required slightly more discrete actions despite lower SIDI-neutral additive cost.

Note: These diagnostics were used as supplemental robustness checks for the SIDI-neutral recourse-audit findings. The design-effect sensitivity interval evaluates whether the raw cost-gap direction was sensitive to conservative variance inflation. Alternative SIDI cutpoints assess whether the direction of the raw burden contrast depended on the prespecified SIDI threshold. The action-count gap illustrates metric dependence and should be interpreted separately from the SIDI-neutral additive cost. SIDI indicates Social Indicators of Disparity Index; DEFF, design effect.

Supplementary Table S14. Exploratory 2022 multi-axis Oaxaca-Blinder decomposition using SIDI-neutral cost.

Equity axis	Raw gap	Explained component	Unexplained residual	Interpretation
SIDI ≥ 2 vs. SIDI 0-1	-0.066	-0.079	+0.013	Primary SIDI residual was small under the neutral cost specification.
Income categories 1-2 vs. 5-6	+0.052	-0.027	+0.079	Strongest 2022 secondary axis signal.
Income median split	+0.010	-0.026	+0.036	Smaller positive income-axis signal.
Age median split	+0.289	+0.254	+0.036	Raw gap was mostly explained by baseline covariate endowments.
Race/ethnicity	+0.017	+0.013	+0.004	Near-null unexplained residual.
Sex	-0.055	-0.060	+0.005	Near-null unexplained residual.

Note: The outcome was SIDI-neutral normalized recourse cost among reachable adults in the 2022 full artifact. Raw gaps are calculated as monitored or disadvantaged group minus reference group differences, as defined for each exploratory contrast. The explained component reflects differences in included Oaxaca-Blinder covariates, whereas the unexplained residual represents the remaining conditional cost difference not accounted for by those covariates. These analyses were exploratory multi-axis scans and were not used to redefine the primary SIDI-neutral estimand. SIDI indicates Social Indicators of Disparity Index.

Supplementary Table S15. Income dose–response Oaxaca-Blinder decomposition using income category 6 as the reference group.

Income level vs. income category 6	Disadvantaged N	Raw gap	Explained component	Unexplained residual [95% CI]	One-sided bootstrap *P* value
Income category 1 vs. 6	905	+0.107	+0.009	+0.098 [+0.008 to +0.176]	*P* = .015
Income category 2 vs. 6	1,622	+0.068	-0.028	+0.097 [+0.011 to +0.176]	*P* = .005
Income category 3 vs. 6	3,028	+0.011	-0.037	+0.048 [-0.013 to +0.108]	*P* = .055
Income category 4 vs. 6	3,810	+0.032	+0.022	+0.010 [-0.034 to +0.061]	*P* = .315
Income category 5 vs. 6	6,498	+0.040	+0.036	+0.004 [-0.032 to +0.036]	*P* = .370

Note: The outcome was SIDI-neutral normalized recourse cost among reachable adults. Income category 6 served as the reference group. Raw gaps are calculated as each income category minus income category 6. The unexplained residual was largest for income categories 1 and 2, attenuated for income category 3, and was near-null for income categories 4 and 5. These analyses were exploratory dose–response diagnostics and should not be interpreted as causal income effects. SIDI indicates Social Indicators of Disparity Index; CI, confidence interval.

Supplementary Table S16. Constraint-relaxation diagnostic based on a 10,000-person high-risk subsample.

Stage	Reachable, %	Unreachable, %	Mean score reduction, low SIDI	Mean score reduction, high SIDI	High-minus-low gap
Stage 0: Unconstrained	99.5	0.5	3.7121	3.3700	-0.3421
Stage 1: C1 immutability locks	61.1	38.9	1.2435	1.2882	+0.0447
Stage 2: C1 + C2 directionality	60.9	39.1	1.2380	1.2817	+0.0437
Stage 3: C1 + C2 + C3 full constraints	60.9	39.1	1.2369	1.2808	+0.0438

Note: This table reports a constraint-relaxation diagnostic based on a 10,000-person high-risk subsample. It evaluates maximum model-score reducibility under staged constraint relaxation and should not be interpreted as the primary recourse reachability estimate. The primary reachability estimate of 10.61% was calculated using the full 2022 high-risk cohort, locked adverse-risk threshold, prespecified action ontology, exact candidate-action generation, sparsity restriction, and minimum-cost optimization. C1 denotes immutability locks, C2 behavioral directionality, and C3 physiological safety floors. SIDI indicates Social Indicators of Disparity Index.

Supplementary Table S17. Primary-artifact action-space compatibility sensitivity.

Action-space specification	Maximum k	Compatible reachable N	Overall compatible reachability	Low-/high-SIDI reachability	Low-/high-income reachability	Weighted mean conditional cost	Weighted SIDI cost gap, high-low	Weighted income cost gap, low-high
BMI only	1	8,533	5.1%	5.2% / 4.1%	2.4% / 9.3%	1.486	0.000	-0.000
BMI only	3	8,533	5.1%	5.2% / 4.1%	2.4% / 9.3%	1.486	0.000	-0.000
Smoking only	1	7,973	4.8%	4.3% / 8.5%	2.5% / 8.2%	0.743	0.000	0.000
Smoking only	3	7,973	4.8%	4.3% / 8.5%	2.5% / 8.2%	0.743	0.000	0.000
BMI + smoking	1	16,506	9.9%	9.5% / 12.5%	4.9% / 17.5%	1.081	-0.101	-0.022
BMI + smoking	2	17,766	10.6%	10.2% / 13.8%	5.5% / 18.6%	1.171	-0.066	0.052
BMI + smoking	3	17,766	10.6%	10.2% / 13.8%	5.5% / 18.6%	1.171	-0.066	0.052
BMI + smoking + binge drinking	1	16,506	9.9%	9.5% / 12.5%	4.9% / 17.5%	1.081	-0.101	-0.022
BMI + smoking + binge drinking	2	17,766	10.6%	10.2% / 13.8%	5.5% / 18.6%	1.171	-0.066	0.052
BMI + smoking + binge drinking	3	17,766	10.6%	10.2% / 13.8%	5.5% / 18.6%	1.171	-0.066	0.052

Note: This sensitivity analysis used the saved primary SIDI-neutral recourse artifact to preserve exact numerical consistency with the main manuscript and positive-control analyses. A row counted an adult as compatible when the saved optimal recourse plan used only the listed action features and no more than the specified maximum k. Reachability percentages use unweighted analytic denominators, matching the main reachability reporting. Conditional costs and cost gaps are survey-weighted among compatible reachable adults. SIDI contrasts compare high SIDI with low SIDI. Income contrasts compare income categories 1-2 with categories 5-6. SIDI indicates Social Indicators of Disparity Index.

Supplementary Table S18. Positive-control simulations for deliberately injected prescriptive inequity.

Scenario	Overall reachability	Low-/high-income reachability	Mean conditional cost	SIDI raw gap	SIDI Oaxaca-Blinder residual	Income raw gap	Income Oaxaca-Blinder residual
Observed primary audit	10.6%	5.5% / 18.6%	1.171	-0.066	+0.013	+0.052	+0.079
PC1: +25% cost for low-income reachable adults	10.6%	5.5% / 18.6%	1.224	-0.001	+0.051	+0.356	+0.384
PC2: +25% cost for high-SIDI reachable adults	10.6%	5.5% / 18.6%	1.218	+0.213	+0.292	+0.139	+0.147
PC3: remove smoking action from low-income optimal plans	9.7%	2.4% / 18.6%	1.186	-0.052	-0.002	+0.320	-0.002

Note: Positive-control simulations used the saved primary SIDI-neutral recourse outputs to deliberately perturb conditional cost or reachable status without retraining the prediction model. PC1 inflated conditional costs by 25% for low-income reachable adults. PC2 inflated conditional costs by 25% for high-SIDI reachable adults. PC3 removed smoking-status optimal actions from low-income adults, thereby testing whether the audit framework detected changes in first-stage reachability and income-stratified accessibility. These simulations demonstrate that the reporting framework responds to deliberately injected prescriptive inequity in reachability, conditional cost, raw gaps, and Oaxaca-Blinder residuals. They should be interpreted as audit-responsiveness stress tests rather than empirical equity estimates. SIDI indicates Social Indicators of Disparity Index.

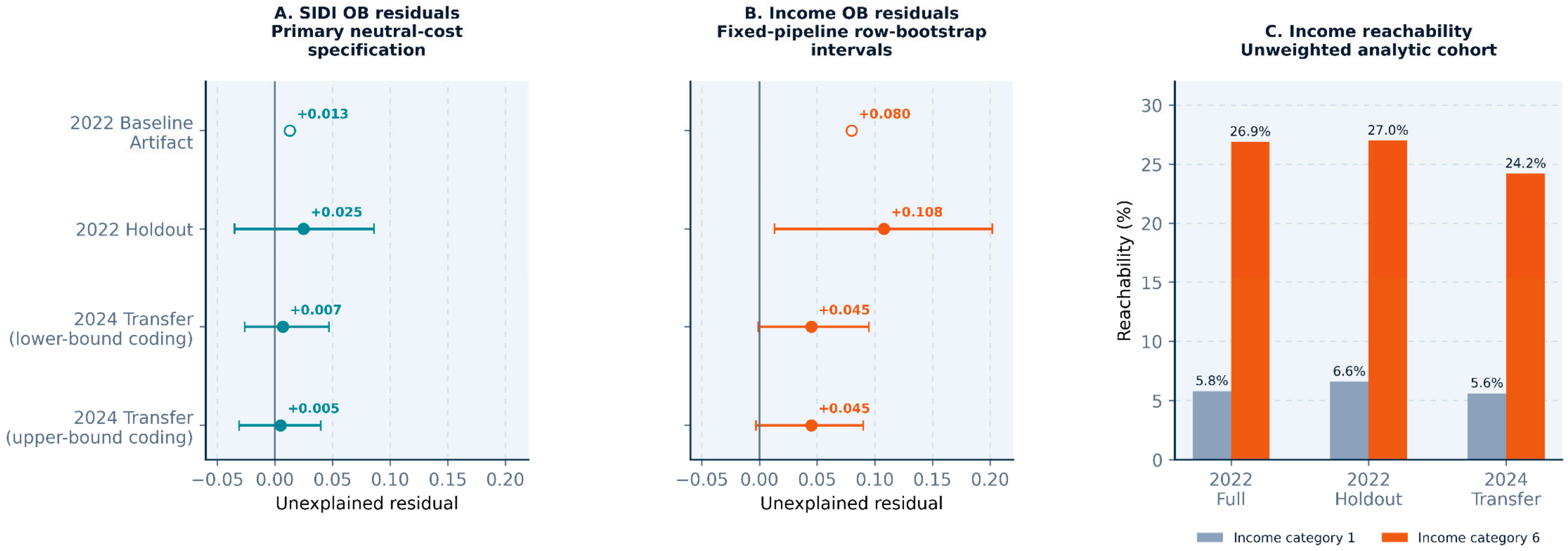

Supplementary Figure S1. Cross-cycle equity-axis comparison of SIDI residuals, income residuals, and income reachability gradients. Panel A shows SIDI Oaxaca–Blinder residuals under the prespecified primary SIDI-neutral additive-cost specification; the 2024 lower- and upper-bound labels refer to missing-component coding, not to low- and high-SIDI populations. Panel B shows income residuals with fixed-pipeline weighted row-bootstrap intervals; those intervals did not preserve STRATA or PSU structure. Panel C consistently applies the primary unweighted analytic-cohort reachability estimand across stages. The verified 2022 baseline values are 5.8% for income category 1 and 26.9% for category 6; the holdout values are 6.6% and 27.0%, and the 2024 values are 5.6% and 24.2%. Survey-weighted income-stratum sensitivities are reported in Supplementary Table S6. SIDI and income are distinct audit axes. OB indicates Oaxaca–Blinder; SIDI, Social Indicators of Disparity Index; PSU, primary sampling unit.

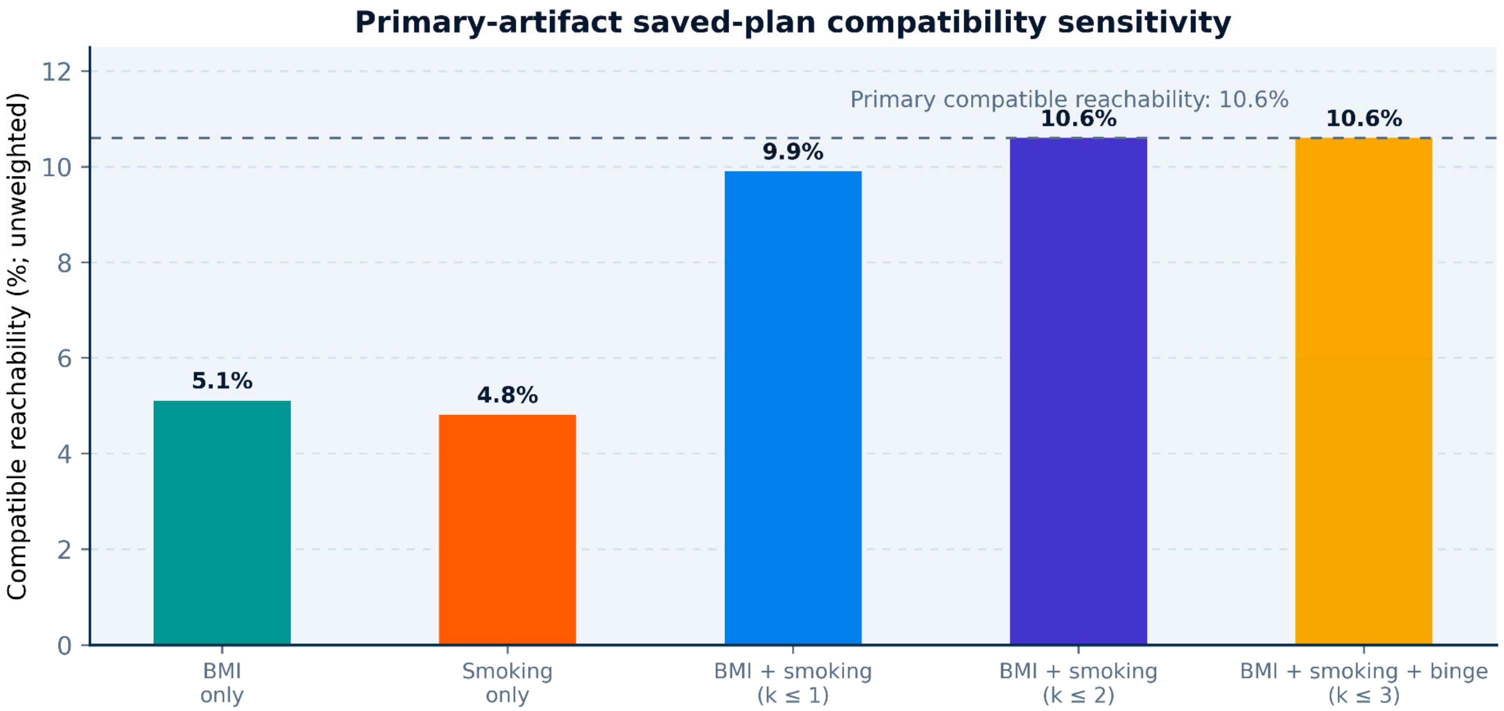

Supplementary Figure S2. Primary-artifact saved-plan compatibility sensitivity in the 2022 full artifact. Compatible reachability uses unweighted analytic denominators. BMI-only and smoking-only saved-plan action spaces yielded 5.1% and 4.8% compatibility. Combining BMI and smoking yielded 9.9% when limited to one action and 10.6% when allowing two actions. Adding binge drinking under $k \leq 3$ did not increase compatibility beyond 10.6%. This diagnostic classifies the actions present in saved optimal plans and does not regenerate or re-optimize recourse under expanded action spaces.
